# Investigating the effects of cytokine biomarkers on HIV incidence: a case study for individuals randomized to pre-exposure prophylaxis vs. control

**DOI:** 10.3389/fpubh.2024.1393627

**Published:** 2024-06-25

**Authors:** Sarah Ogutu, Mohanad Mohammed, Henry Mwambi

**Affiliations:** ^1^School of Mathematics, Statistics and Computer Science, University of KwaZulu-Natal, Pietermaritzburg, South Africa; ^2^School of Nursing and Public Health, University of KwaZulu-Natal, Pietermaritzburg, South Africa

**Keywords:** cytokine biomarkers, HIV incidence, pre-exposure prophylaxis, stepwise Cox PH, Kaplan–Meier

## Abstract

**Introduction:**

Understanding and identifying the immunological markers and clinical information linked with HIV acquisition is crucial for effectively implementing Pre-Exposure Prophylaxis (PrEP) to prevent HIV acquisition. Prior analysis on HIV incidence outcomes have predominantly employed proportional hazards (PH) models, adjusting solely for baseline covariates. Therefore, models that integrate cytokine biomarkers, particularly as time-varying covariates, are sorely needed.

**Methods:**

We built a simple model using the Cox PH to investigate the impact of specific cytokine profiles in predicting the overall HIV incidence. Further, Kaplan-Meier curves were used to compare HIV incidence rates between the treatment and placebo groups while assessing the overall treatment effectiveness. Utilizing stepwise regression, we developed a series of Cox PH models to analyze 48 longitudinally measured cytokine profiles. We considered three kinds of effects in the cytokine profile measurements: average, difference, and time-dependent covariate. These effects were combined with baseline covariates to explore their influence on predictors of HIV incidence.

**Results:**

Comparing the predictive performance of the Cox PH models developed using the AIC metric, model 4 (Cox PH model with time-dependent cytokine) outperformed the others. The results indicated that the cytokines, interleukin (IL-2, IL-3, IL-5, IL-10, IL-16, IL-12P70, and IL-17 alpha), stem cell factor (SCF), beta nerve growth factor (B-NGF), tumor necrosis factor alpha (TNF-A), interferon (IFN) alpha-2, serum stem cell growth factor (SCG)-beta, platelet-derived growth factor (PDGF)-BB, granulocyte macrophage colony-stimulating factor (GM-CSF), tumor necrosis factor-related apoptosis-inducing ligand (TRAIL), and cutaneous T-cell-attracting chemokine (CTACK) were significantly associated with HIV incidence. Baseline predictors significantly associated with HIV incidence when considering cytokine effects included: age of oldest sex partner, age at enrollment, salary, years with a stable partner, sex partner having any other sex partner, husband's income, other income source, age at debut, years lived in Durban, and sex in the last 30 days.

**Discussion:**

Overall, the inclusion of cytokine effects enhanced the predictive performance of the models, and the PrEP group exhibited reduced HIV incidences compared to the placebo group.

## 1 Introduction

HIV continues to be a serious worldwide health concern, with South Africa having the world's highest HIV epidemic, with an estimated 8.45 million people living with HIV (1). The primary mode of transmission in this endemic setting is heterosexual intercourse, and women 18–40 years account for more than 60% of new infections where the young women bear the greatest burden (2). It is well known that sex workers are at a greater risk of HIV acquisition (3). Significant effort has been made in South Africa over the last decade to search for new technologies that prevent sexually transmitted HIV infections in women such as the pre-exposure prophylaxis (PrEP) products. Initiatives have been undertaken to scale up education and access to these products for example the tenofovir gel an antiretroviral microbicide that can be applied to the vagina or rectum with intentions of reducing the acquisition of HIV (4).

The initial stages of HIV infection are characterized by inflammation and profound immune dysregulation in the gut mucosa (5, 6) and genital inflammation at this stage also correlates with an increased plasma viral load (7). Taken together, inflammation is a key mediator of HIV pathogenesis. The levels of inflammatory cytokines and chemokines, which signal the presence of infection and recruit activated immune cells to the mucosa, are frequently used as biomarkers of inflammation in the female reproductive tract (FRT) (8). As such, we hypothesize it might be expected that elevated mucosal cytokines would be correlated with increased rates of HIV acquisition. The increased levels of pro-inflammatory cytokine is associated with increased rates of HIV acquisition (9) and cytokine profile is a strong predictor of subsequent HIV acquisition. Understanding the interplay between cytokine biomarkers and HIV incidence by identifying specific cytokine profiles associated with increased or decreased HIV susceptibility is crucial for optimizing PrEP strategies.

Cytokines serve a vital role in maintaining immune system homoeostasis (10), and HIV infection causes dysregulation of the cytokine profile (11). Changes in the cytokine signature directly affect HIV disease progression (12), with an intense cytokine “storm” during acute HIV infection (13). T-helper type 1 (Th1) cytokines such as interleukin (IL)-2 and antiviral interferon (IFN)-gamma are generally decreased during HIV infection, whereas T-helper type 2 (Th2) cytokines such as IL-4, IL-10, pro-inflammatory cytokines (IL-1, IL-6, IL-8) and tumor necrosis factor (TNF)-alpha are increased (10). IFN-alpha, IFN-beta, and IL-16 are HIV-suppressive cytokines that inhibit HIV replication in T cells while IFN-gamma, IL-4, and granulocyte-macrophage colony-stimulating factor, for example, have been demonstrated to have both inhibitory and stimulatory effects on HIV (14).

In clinical research, it is a common phenomenon for covariate data to be collected longitudinally and for the covariates to change over time during the follow up period. For example, patients in a clinical trial to asses the safety and effectiveness of tenofovir gel, a vaginal microbicide in sexually active women at risk for HIV, cytokine profiles were measured repeatedly up to infection or until censorship (4). In many instances, while examining the relationship between time to HIV infection and covariate(s), investigators will only consider the baseline covariates, leaving out covariates that change over time hence failing to consider the relation of the survival outcome as a function of the change of the time dependent covariates (15). It appears natural and suitable to use time-varying covariate information in an appropriate statistical model. The Cox PH model can be used to link survival times with either fixed covariates whose values remain constant during the follow-up period or predictor variables that fluctuate over time (16). The mentioned covariates can be dealt with as a time dependent covariates into the Cox PH model or incorporated as a derived longitudinal variables as further elaborated in the Section 2.2.

A previous analysis was conducted by Abdool Karim et al. (4) and Mansoor et al. (17) to investigate the effectiveness, safety and adherence in the CAPRISA 004 tenofovir gel microbicide trial. They used Proportional Hazards (PH) regression model to calculate the hazard ratios while adjusting for potentially important baseline covariates (age, site, anal sex history, contraceptive method, HSV-2, antibody status and condom use). They reported a hazard ratio of 0.63 (CI: 0.42,0.94, *p* = 0.025). In their analysis they did not include cytokine profile neither did they report on significant baseline covariates associated with HIV incidence. Masson et al. (18) used the same dataset to investigate whether genital inflammation influenced HIV acquisition in women. Their study selected 12 cytokines for their analysis. They employed conditional logistic regression and unsupervised hierarchical clustering in their statistical analysis.

Naranbhai et al. (19) investigated the role of immune activation in HIV acquisition in the CAPRISA 004 trial. They selected 13 cytokines and used logistic regression and principal component analysis (PCA) in their statistical analysis. On the other hand, Ngcobo et al. (20), in their study examining whether pre-infection plasma cytokine concentrations predicted the rate of HIV disease progression in the same study cohort, considered all 48 cytokines. They used linear regression to assess the impact of each cytokine on viral load (VL) and the CD4:CD8 ratio in both bivariate and multivariable models, adjusting for age, contraceptive use, HSV-2 status at baseline, study site, and study arm at randomization. Ignacio et al. (21) used the Sabes dataset and LASSO machine learning algorithms to study how dynamic immune markers predict HIV acquisition and strengthen associations with sociobehavioral factors related to HIV exposure. They selected 10 cytokines for their analysis. Other studies (22, 23) that have utilized CAPRISA 004 data set to investigate HIV progression, did not include time varying cytokine profile as a covariate in their analysis. To the best of our knowledge, cytokine profile as a time-varying covariate or as derived covariate has not been used with baseline covariate in previous studies to identify significant predictors of HIV incidence.

This study therefore, seeks to investigate the effect of time-varying cytokine biomarkers in determining significant predictors of HIV incidence among individuals randomized to PrEP vs. control exposure. We achieved that by building a series of Cox PH models that include different forms of the covariates that change over time and further asses the overall effectiveness of the tenofovir treatment by comparing the two groups using Kaplan–Meier estimator and survival curves. The variations in individual immune responses, particularly in cytokine profiles, may influence the efficacy of PrEP therefore this research aims to contribute to the development of personalized PrEP interventions tailored to individual immune responses.

## 2 Materials and methods

### 2.1 Dataset

The data was accessed from Center for the Aids Programme of Research in South Africa (CAPRISA 004) (4), a two arm double blinded randomized trial, placebo and tenofovir group conducted on HIV negative and sexually active women aged 18–40 years in South Africa for a period of 30 months; 18 months Accrual period and 12 months follow up. It was conducted between May 2007 and March 2010, and the dataset consist of survival and longitudinal data. The variables considered in this study were baseline characteristics and longitudinally measured cytokine profiles as described in the Supplementary Table S1.

#### 2.1.1 Cytokine measurement

**Figure 1:**
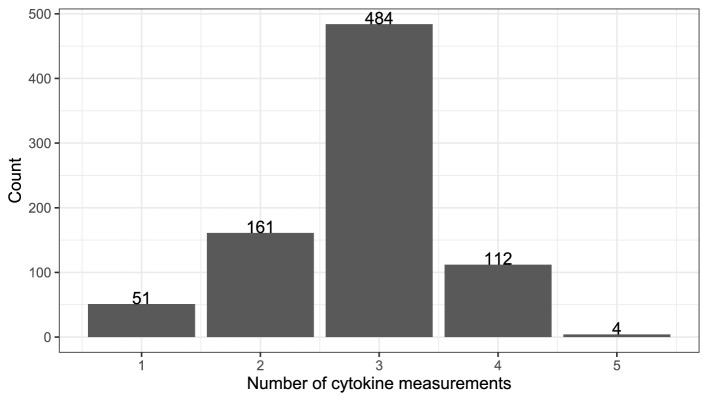
Total count for the frequency of cytokine profile measurements.

Plasma samples and cervicovaginal lavage specimens from cases and control were collected and stored for assessment. There were a total of 48 cytokines from 812 (tenofovir group = 405, placebo group = 407) women with 96 HIV infections (tenofovir group = 37, placebo = 59). The measurements were taken at irregular follow up times as shown in Figure 1 where majority of patients had their cytokine measurements recorded three times during the course of study. The average interval between the first and second cytokine measurements was 12 months, while the interval between subsequent cytokine measurements was 6 months.

#### 2.1.2 Data pre-processing

**Figure 2:**
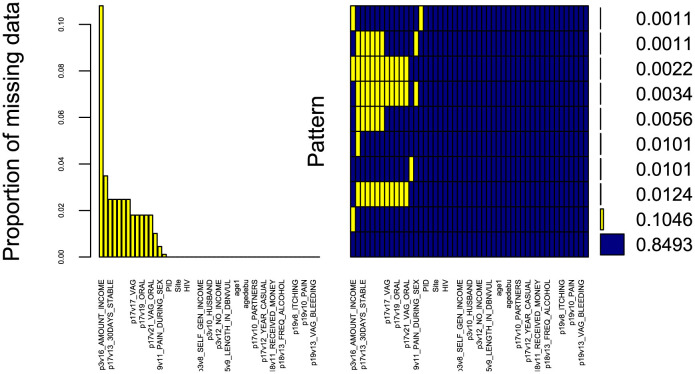
Missing data aggregation plot. Proportion of missing values for all variables in the dataset, sorted by decreasing order **(left)**. Combinations of missing values **(right)**: yellow squares in a matrix entry denote the presence of missing values for the variable associated to the column in the samples corresponding to the row; the bars on the right show the cardinality of each set of points. The *x*-axis displays the variable names (not all variables are displayed due to limited space).

The data underwent pre-processing to prepare it for subsequent analysis. The pre-processing steps involved eliminating variables with excessive missing values (Figure 2) i.e with more than 50% missingness and very small frequency percentages for the levels of some categorical variables. Additionally, in our efforts to enhance the robustness of our statistical analysis, we appropriately combined certain levels of categorical variables and renamed the strings. This step is crucial because a categorical variable with too many levels can compromise the model's performance due to small frequencies in some of the levels (24). Moreover, variables with only one level fail to positively impact the model due to very low variation, while levels that rarely occur have minimal chance of significantly affecting the model fit (25). These adjustments ensure that our analysis accurately captures the relationships within the data. Furthermore, Figure 2 demonstrates the completeness of our dataset, with almost 84.93% of variables containing no missing information, 10.46% missing income value data, and the remaining variables displaying other missing patterns.

The data preparation and the statistical analysis was done using the *R* version (R-4.3.2). The R code file for this analysis is available in the Supplementary Table S2. As a result of the pre-processing step, 24 baseline characteristics and 48 cytokines covariates were used as initial variables at the start of the analysis. The categorical variables were summarized using frequency and percentages depicted in the Supplementary Table S2. The patient baseline characteristics in relation to HIV status and treatment group is summarized in Table 1, where the number of years with stable partner (*p* = 0.034) and the patients receiving income from husband (*p* = 0.026) were significantly associated with HIV status and treatment group. The statistical analysis was conducted on complete cases only in two stages; the first is survival analysis on baseline covariates without the cytokine covariates effects then survival analysis when including the cytokine covariate effects. Cytokine variable profiles are time-varying covariates since they change over time through the follow-up period. Therefore, the cytokine information was included in three ways; firstly we averaged all measurements throughout the follow-up time to better capture their average effects, secondly we took the difference between the last and first measurement to model the effect of change and lastly we treated the cytokine as a time-dependent covariate.

**Table 1:** Summary description of patient's baseline characteristics stratified by HIV status and treatment group.

**Variables**	**0:Placebo**	**1:Placebo**	**0:Tenofovir**	**1:Tenofovir**	***p***
*n*	327	51	353	33	
Treat = Tenofovir (%)	0 (0.0)	0 (0.0)	353 (100.0)	33 (100.0)	<0.001
Site = Vulindlela (%)	215 (65.7)	33 (64.7)	239 (67.7)	21 (63.6)	0.917
months (mean [SD])	19.25 (5.86)	8.54 (5.52)	19.25 (5.57)	10.86 (7.28)	<0.001
Partner live together = No (%)	288 (88.1)	46 (90.2)	298 (84.4)	30 (90.9)	0.367
Highest education (%)					0.262
High school	289 (88.4)	46 (90.2)	311 (88.1)	26 (78.8)	
Primary	12 (3.7)	1 (2.0)	21 (5.9)	4 (12.1)	
Tertiary	26 (8.0)	4 (7.8)	21 (5.9)	3 (9.1)	
Self generated Income = Yes (%)	19 (5.8)	1 (2.0)	19 (5.4)	0 (0.0)	0.361
Salary = Yes (%)	38 (11.6)	6 (11.8)	36 (10.2)	6 (18.2)	0.565
Husband's income = Yes (%)	49 (15.0)	4 (7.8)	39 (11.0)	9 (27.3)	0.026
Social grants = Yes (%)	257 (78.6)	42 (82.4)	283 (80.2)	24 (72.7)	0.701
Other income source = Yes (%)	30 (9.2)	5 (9.8)	27 (7.6)	5 (15.2)	0.499
Income amount = <R10001 (%)	293 (89.6)	46 (90.2)	321 (90.9)	29 (87.9)	0.908
Years in Durban (mean [SD])	16.49 (9.05)	17.94 (7.94)	17.23 (9.46)	15.48 (8.55)	0.466
Age at enrollment (mean [SD])	24.02 (5.11)	22.63 (3.55)	24.60 (5.37)	23.64 (4.74)	0.053
Marital status (%)					0.761
Casual	7 (2.1)	2 (3.9)	6 (1.7)	0 (0.0)	
Married	20 (6.1)	2 (3.9)	25 (7.1)	1 (3.0)	
Stable & Casual	11 (3.4)	4 (7.8)	16 (4.5)	2 (6.1)	
Stable	289 (88.4)	43 (84.3)	306 (86.7)	30 (90.9)	
Age at debut (mean [SD])	6.53 (2.03)	6.16 (1.86)	6.43 (2.24)	6.24 (1.92)	0.618
Number of partners (mean [SD])	3.53 (12.45)	3.06 (3.16)	3.12 (6.50)	2.45 (1.70)	0.893
Years with stable partner (mean [SD])	1.02 (0.21)	1.06 (0.31)	1.06 (0.28)	1.18 (1.04)	0.034
Years with casual partner (mean [SD])	0.77 (6.51)	0.31 (0.73)	0.56 (4.50)	0.15 (0.57)	0.865
Stable partners in 30 days (mean [SD])	0.99 (0.11)	0.98 (0.14)	0.99 (0.13)	1.00 (0.00)	0.867
Casual partners in 30 days (mean [SD])	0.17 (1.23)	0.12 (0.38)	0.41 (4.20)	0.12 (0.33)	0.712
Times sex in 30 days (mean [SD])	8.71 (10.19)	7.20 (4.85)	8.91 (8.99)	7.58 (6.52)	0.573
Age of oldest sex partner (mean [SD])	27.69 (6.30)	26.24 (3.82)	28.20 (6.75)	26.88 (5.02)	0.149
Sex partner have other partner (%)					0.228
No	53 (16.2)	2 (3.9)	56 (15.9)	5 (15.2)	
Don't know	199 (60.9)	35 (68.6)	232 (65.7)	21 (63.6)	
Yes	75 (22.9)	14 (27.5)	65 (18.4)	7 (21.2)	
Frequency of condom use = Occasionally (%)	227 (69.4)	37 (72.5)	254 (72.0)	20 (60.6)	0.533
Vaginal abnormal discharge = Yes (%)	97 (29.7)	16 (31.4)	114 (32.3)	17 (51.5)	0.085

0: HIV negative, 1: HIV positive.

### 2.2 Statistical methods

Four separate Cox PH models were fitted in an increasing complexity based on how the cytokine effects are included. Model 1 (Equation 8): Cox regression model with baseline variables only, model 2 (Equation 9): Cox regression model with baseline variables plus cytokine effects using the mean value of the cytokine measurements as covariate, model 3 (Equation 10): Cox regression model with baseline variables plus cytokine effects using the difference between the last observed cytokine value and the first value as covariate in the model and model 4 (Equation 11): Cox regression model with baseline variables plus time dependent cytokine effects.

#### 2.2.1 Kaplan–Meier survival curves

The Kaplan–Meier estimator is a non-parametric statistic that is used to estimate the survival function based on lifetime data (26). The estimate is frequently used in medical research to examine recovery rates, likelihood of deaths and whether or not a treatment was effective. Furthermore, it is used to compare two groups of subjects, the control group and treatment group (27). The Kaplan–Meier survival curve is a graphical representation of the survival function defined as the probability of surviving in a given length of time while considering time in many small intervals (28).

To estimate the survival function *S*(*t*) (the probability that life is longer than *t*), we consider survival time *t*_*i*_ = *t*_1_, *t*_2_, ..., *t*_*n*_ including censored observations (ordered by increasing observation) of a group of n subjects. The proportion of individuals, *S*(*t*), who survive after any follow up time *t*_*i*_ is estimated by (Equation 1)


(1) $$\begin{array}{l} S\left(t\right)=\underset{t_{i}<t}{\prod }\frac{n_{i}-d_{i}}{n_{i}}=\underset{t_{i}<t}{\prod }\left(1-\frac{d_{i}}{n_{i}}\right) \end{array}$$

where *t*_*i*_ is the largest survival time less than or equal to *t*, *n*_*i*_ is the number of individuals uninfected just before time *t*_*i*_ (the *i*^*th*^ ordered survival time) and *d*_*i*_ denotes the number who got HIV infection at time *t*_*i*_ (29). *S*(*t*_0_) = 1 before the first infection of HIV. The survival *S*(*t*) at time *t*_*i*_ given the number of infections *d*_*i*_ and the number of uninfected patients *n*_*i*_ just before *t*_*i*_ is given by (Equation 2),


(2) $$\begin{array}{l} S\left(t_{i}\right)=\frac{n_{i}-d_{i}}{n_{i}}\times S\left(t_{i-1}\right). \end{array}$$

Maximum likelihood estimation of the discrete hazard function *h*_*i*_, (the probability of an individual experiencing an event at time *t*_*i*_), yields the Kaplan–Meier estimator as shown (Equation 3),


(3) $$\hat{S}(t)=\prod_{i:t_{i}\leq t}\left(1-{\hat{h}}_{i}\right)=\prod_{i:t_{i}\leq t}\left(1-\frac{d_{i}}{n_{i}}\right).$$

Moreover, The Kaplan–Meier estimator is a statistic, and its variance is approximated by numerous estimators such as Greenwoods's formula (30) that gives (Equation 4),


(4) $$\hat{Var}\left(\hat{S}(t)\right)=\hat{S}{\left(t\right)}^{2}\sum_{i:t_{i}\leq t}\frac{d_{i}}{n_{i}(n_{i}-d_{i})}$$

The log-Rank test: Is used to compare the hazards between two groups or more by testing the null hypothesis that the probability of an event at any time point between the two or more populations do not differ. Thus, log-rank test compares the survival function of the two groups (27). The null hypothesis will be rejected if the *p*-value is <0.05.

#### 2.2.2 Stepwise Cox proportional hazard model (Cox PH)

Stepwise Cox proportional hazards regression is a method of selecting a subset of relevant variables for a Cox regression model from a larger set (31). Cox PH is the most widely used statistical method for analyzing the time-to-event data (16). The Cox PH model assesses the impact of multiple factors on survival simultaneously. Essentially, it enables one to investigate how specified predictors influence the rate of a specific event happening such as infection or death at a given point in time (32). This rate is commonly known as hazard rate.

In order to evaluate the association of the baseline and cytokine effects covariate and survival time, consider sample size *n* from sample *k* = 1, 2, ..., *n* and *C*_*k*_ = (*C*_*k*1_, *C*_*k*2_, ..., *C*_*kp*_) is a vector of *p* covariates (baseline plus cytokine effect covariates) of the different models. The *k*^*th*^ patient survival data can be represented by (*T*_*k*_, θ_*k*_, *C*_*k*_), where *T*_*k*_ and θ_*k*_ are the survival time and censor status, respectively. Mathematically, the general Cox PH (33) in Equation 5 is represented as


(5) $$\begin{array}{l} h_{k}\left(t;C_{k}\right)=h_{0}\left(t\right)e^{\beta^{\prime }C_{k}} \end{array}$$

where β is the parameter vector of the regression coefficients and **C_*k*_** is the covariate (baseline and cytokine effects) vector. *h*_0_(*t*) is an unspecified baseline hazard function that corresponds to the value of the hazard if all *C*_*k*_ are equal to zero. The hazard ratio for two patients (Equation 6), *k* and *i* is


(6) $$\begin{array}{l} \frac{h_{k}\left(t;C_{k}\right)}{h_{i}\left(t;C_{i}\right)}=\frac{e^{\beta^{\prime }C_{k}}}{e^{\beta^{\prime }C_{i}}} \end{array}$$

and is independent of time *t*. Cox PH model parameters are estimated by the maximum partial likelihood method given below (Equation 7);


(7) $$L(\beta )=\prod_{r\in E}\frac{e^{\beta^{T}}C_{r}}{\;\sum_{i\in R_{r}}e^{\beta^{T}}C_{r}}$$

where *E* is the indices of the HIV infection and *R*_*r*_ represent vector of indices for individuals at risk at time *t*_*r*_.

The stepwise Cox proportional hazards regression method adds or removes predictor variables from the model based on some criteria, such as the Akaike information criterion (AIC) or the Bayesian information criterion (BIC) (34). The AIC and BIC are measures of how well the model fits the data, and they penalize models that have too many parameters. The lower the AIC or BIC, the better the model (35). The stepwise Cox PH regression method can be performed using different methods, such as forward selection, backward elimination, bidirectional selection, or score selection (36). Forward selection starts with an empty model and adds one variable at a time until it reaches a stopping criterion, such as a minimum AIC or BIC value. Backward elimination starts with a full model and removes one variable at a time until it reaches a stopping criterion. Bidirectional selection starts with an empty model and adds one variable at a time in both directions until it reaches a stopping criterion. Score selection starts with an empty model and adds one variable at a time based on its score in terms of AIC or BIC.

We used the packages *StepReg* (37) to implement stepwise regression, *Survival* and *survminer* to implement Cox PH model in R. The specific Cox PH models for model 1, model 2, model 3, and model 4, as described above, are formulated as follows:

#### 2.2.3 Model 1

The model that includes the baseline covariates only. We call this the naive Cox PH model. The assumption is that regression parameters remain constant over time (38). Consequently, the hazard ratio for any two individuals remains constant over time. The Model is given by,


(8) $$\begin{array}{l} h_{k}\left(t;X_{k}\right)=h_{0}\left(t\right)\times exp\left(\beta^{\prime }X_{k}\right) \end{array}$$

with *h*_0_(*t*) as the baseline hazard function, β is the vector of regression coefficients for baseline covariates *X*_*k*_.

#### 2.2.4 Model 2

The model that includes the baseline covariates plus cytokine effects using the mean value of the cytokine measurements as the covariate. Here the derived cytokine is the average of all the cytokine measurements for an individual patient recorded at different follow up time. It models the average effect of the time-varying cytokine covariate (15). The Cox model is;


(9) $$\begin{array}{l} h_{k}(t;X_{k},G_{k})=h_{0}(t)\times exp\left(\beta^{\prime }X_{k}+\delta^{\prime }{\bar{G}}_{k}\right)=h_{0}(t)\times exp\\ \;\left(\beta^{\prime }X_{k}+\sum_{j=1}^{v}\delta_{j}{\bar{G}}_{kj}\right) \end{array}$$

where *h*_0_(*t*) is the baseline hazard function, β is the regression coefficient vector for time invariant covariates *X*_*k*_, ${Ḡ}_{kj}=\frac{1}{m_{kj}}\overset{m_{kj}}{\underset{r=1}{\sum }}G_{krj}$ for *r* = 1, 2, ..., *m*_*kj*_, represents the average value of the cytokine level measured longitudinally for the *k*^*th*^ subject with *m*_*kj*_ observations for cytokine *j* (*j* = 1, 2, ..., *v*). The scalar δ_*j*_∈ℝ is the parameter that links the average to the hazard.

#### 2.2.5 Model 3

The model that includes the baseline covariates plus cytokine effects using the difference between the last observed cytokine value and the first value as the covariate in the model. It models the effect of change in the cytokine covariates (39).


(10) $$\begin{array}{l} h_{k}(t;X_{k},D_{k})=h_{0}(t)\times exp\left(\beta^{\prime }X_{k}+\delta^{\prime }D_{k}\right)=h_{0}(t)\times exp\\ \;\left(\beta^{\prime }X_{k}+\sum_{j=1}^{v}\delta_{j}D_{kj}\right) \end{array}$$

where *h*_0_(*t*) is the baseline hazard function, β is the regression coefficient vector for the time invariant covariates *X*_*k*_, *D*_*kj*_ = [*G*_*k*_*m*__*kj*__−*G*_*k*1*j*_] represents the difference between the last and first cytokine levels observed longitudinally for the *k*^*th*^ subject, with *m*_*kj*_ measurements for cytokine *j* (*j* = 1, 2, ..., *v*). The scalar δ_*j*_∈ℝ is the parameter that links the change to the hazard. The model answers the question whether a big or small change in cytokine has an effect on HIV acquisition.

#### 2.2.6 Model 4

The model that includes the baseline covariates plus time dependent cytokine effects. When a covariate changes over time throughout the follow-up period, this is referred to as time-varying/time dependent covariate (40). For this it is critical to structure the data in a counting process format. We code the time-dependent covariate using time intervals (41). The hazard is assumed to be proportional to the instantaneous probability of an event at a specific time conditional on the variables at that time (42). The interpretation of the results of this approach is more complicated than a naïve baseline approach as the covariate information changes over time. Here we consider sample size *n* subjects, consisting of [*T*_*k*_, θ_*k*_, [*G*_*k*_(*t*), 0 ≤ *t* ≤ *T*_*k*_], *k* = 1, 2, ..., *n*], *T*_*k*_ is the time-to event for the *k*th subject, θ_*k*_ is the event indicator and *G*_*k*_(*t*) is the time varying covariate. The Cox PH model becomes,


(11) $$\begin{array}{l} h(t;X_{k},G_{k})=h_{0}(t)\times exp\left(\beta^{\prime }X_{k}+\delta^{\prime }G_{k}(t)\right)=h_{0}(t)\times exp\\ \;\left(\beta^{\prime }X_{k}+\;\sum_{j=1}^{v}\delta_{j}G_{kj}(t)\right) \end{array}$$

where *h*_0_(*t*) is the baseline hazard function, β is the regression coefficient vector for time invariant covariates, *G*_*kj*_(*t*) = [*G*_*k*1*j*_(*t*), *G*_*k*2*j*_(*t*), ..., *G*_*k*_*m*__*kj*__(*t*)] is a set of covariates for the number of longitudinal measures *m*_*kj*_ for the *k*^*th*^ subject of cytokine *j* (*j* = 1, 2, ..., *v*). The scalar δ_*j*_∈ℝ represent the parameter that links the time dependent covariates to the hazard.

## 3 Results

### 3.1 Kaplan–Meier survival curves analysis

**Figure 3:**
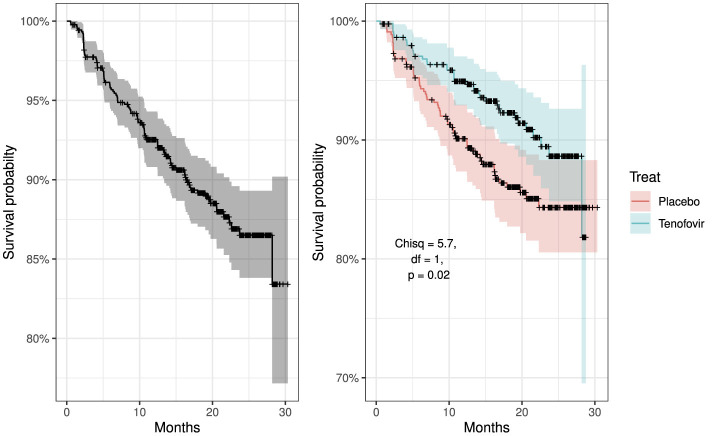
Kaplan–Meier survival curves: left panel showing overall survival curve for all participants and right panel compares overall survival curves by treatment groups, placebo vs. tenofovir participants.

Figure 3 shows the overall survival curve over time and the overall survival comparison between the two treatment group. It is clear that patients from Tenofovir treatment arm have a better chance of surviving (less probability of HIV acquisition) more than the patients from placebo group. The placebo curve has a steeper slope indicating a higher HIV infection rate, therefore a worse survival prognosis. The curve have plateaus from 24th month indicating no change in survival. The curves comparing the two treatment cross in the first few months and consistently separate afterwards. The log-rank test performed gives χ^2^ = 5.7, *df* = 1 and *p*-value = 0.02. Since *p*-value is <0.05 we reject the null hypothesis to conclude that there is sufficient evidence indicating that the two treatment groups are significantly different in terms of survival.

### 3.2 Stepwise Cox proportional hazard analysis

The results of the survival problem based on the effects of cytokine biomarkers (mean, difference, and time dependent effect) were obtained. As a first step, we employed the stepwise regression using the *stepwiseCox* function. Within the function we specified the following arguments; model selection procedure to be bidirectional, model selection metric as the AIC, significance level of entry and exit value in the model as 0.15 and model approximation method as Efron. Bidirectional selection procedure is the appropriate since it adds variables in both directions. Moreover, backward selection produces same results as bidirectional while forward selection produces results with more covariates and larger AIC. The model selection criterion AIC was used to determine the order in which effects enter and leave at each step of the specified model selection procedure (bidirection). The value 0.15 is a commonly used p value threshold which is a statistical significance level that a predictor variable must meet to be included or to stay in the model. Several approximation methods have been proposed to handle tied events in cox regression such as Breslow, Efron, and Exact (methods of obtaining Cox partial likelihood estimate of the baseline hazard function). However, the Efron method performs better in terms of time, fit statistics, and differences in parameters estimates (43). We then tested the Cox PH assumption of the selected covariates using Schoenfeld residuals test (44) by applying the *cox*.*zph* function. The analysis of the results for model 1-4 are shown in Tables 2–5.

**Table 2:** Multivariable Cox PH results for predictors of HIV survival among women aged 18–40 years (model 1).

**Variable**	**HR (SE)**	**95% CI**	***p*-value**
Age at enrollment	0.949 (0.026)	(0.902, 0.998)	0.042*
Treatment group			
Placebo	1.000		
Tenofovir	0.6286 (0.225)	(0.405, 0.977)	0.039*
Self generated income			
No	1.000		
Yes	0.225 (1.013)	(0.031, 1.634)	0.140
Vaginal abnormal discharge			
No	1.000		
Yes	1.431 (0.225)	(0.920, 2.226)	0.112
Salary			
No	1.000		
Yes	1.658 (0.322)	(0.882, 3.116)	0.116
Sex partner have other partner			
No	1.000		
Don't know	1.849 (0.403)	(0.840, 4.072)	0.127
Yes	2.280 (0.438)	(0.966, 5.383)	0.060

^*^p-value <0.05.

**Table 3:** Multivariable Cox PH results for predictors of HIV survival among women aged 18–40 years (model 2).

**Variable**	**HR (SE)**	**95% CI**	***p*-value**
Age of oldest sex part	0.945 (0.026)	(0.898, 0.995)	0.031*
Treatment group			
Placebo	1.000		
Tenofovir	0.486 (0.253)	(0.296, 0.798)	0.004**
M-CSF	1.004 (0.001)	(1.002, 1.006)	0.000***
Salary			
No	1.000		
Yes	2.474 (0.358)	(1.227, 4.987)	0.011*
Frequency of condom use			
Always	1.000		
Occasionally	1.543 (0.272)	(0.905, 2.632)	0.111
Age at debut	0.884 (0.069)	(0.772, 1.012)	0.073
Number of stable partner (past year)	1.480 (0.188)	(1.025, 2.138)	0.037*
Vaginal abnormal discharge			
No	1.000		
Yes	1.513 (0.249)	(0.930, 2.463)	0.096
Sex partner have other partner			
No	1.000		
Don't know	2.948 (0.441)	(1.241, 7.001)	0.014*
Yes	2.613 (0.492)	(0.996, 6.854)	0.051
Self generated income			
No	1.000		
Yes	0.261 (1.040)	(0.034, 2.005)	0.197
MIG	1.000 (0.000)	(1.000, 1.000)	0.001**
MIP-1B	1.001 (0.000)	(1.001, 1.001)	0.000***
MCP-1	0.991 (0.005)	(0.982, 1.000)	0.053
SCF	1.113 (0.017)	(1.077, 1.151)	0.000***
IL-12P70	0.977 (0.005)	(0.968, 0.986)	0.000***
IL-16	0.996 (0.002)	(0.992, 1.000)	0.034*
MIF	1.000 (0.000)	(1.000, 1.0000)	0.001***
B-NGF	0.807 (0.095)	(0.670, 0.973)	0.024*
SCGF-B	0.995 (0.000)	(0.993, 0.999)	0.000***
TNF-A	1.018 (0.005)	(1.009, 1.027)	0.000***
IL-17A	0.979 (0.007)	(0.965, 0.992)	0.002**
MCP-3	0.982 (0.011)	(0.961, 1.004)	0.110
CTACK	1.039 (0.012)	(1.016, 1.063)	0.001***
IL-10	1.049 (0.018)	(1.013, 1.086)	0.007**
IL-5	1.106 (0.049)	(1.004, 1.218)	0.041*
G-CSF	1.000 (0.000)	(1.000, 1.000)	0.034*
IL-3	0.989 (0.004)	(0.982, 0.996)	0.003**
IL-2RA	1.023 (0.013)	(0.997, 1.049)	0.085
IFN-A2	1.028, (0.011)	(1.006, 1.050)	0.012*

^*^p-value <0.05, ^**^p-value <0.01, ^***^p-value <0.001.

**Table 4:** Multivariable Cox PH results for predictors of HIV survival among women aged 18–40 years (model 3).

**Variable**	**HR (SE)**	**95% CI**	***p*-value**
Age of oldest sex partner	0.946 (0.023)	(0.903, 0.990)	0.017*
Sex partner have other partner			
No	1.000		
Don't know	2.6235 (0.441)	(1.106, 6.223)	0.029*
Salary			
No	1.000		
Yes	1.887 (0.330)	(0.988, 3.607)	0.055
Yes	3.991 (0.487)	(1.535, 10.373)	0.005**
Treatment group			
Placebo	1.000		
Tenofovir	0.652 (0.238)	(0.409, 1.039)	0.072
Husband's income			
No	1.000		
Yes	1.901 (0.316)	(1.023, 3.534)	0.042*
Self generated income			
No	1.000		
Yes	0.267 (1.028)	(0.036, 2.003)	0.199
IL-1RA	1.000 (0.000)	(1.000, 1.000)	0.004**
MIF	1.000 (0.000)	(1.000, 1.000)	0.000***
B-NGF	0.896 (0.026)	(0.851, 0.943)	0.000***
CTACK	1.022 (0.005)	(1.013, 1.032)	0.000***
IL-5	0.923 (0.023)	(0.882, 0.966)	0.001***
IL-2	1.106 (0.033)	(1.037, 1.179)	0.002**
IL-1A	1.000 (0.000)	(0.999, 1.000)	0.106
TRAIL	0.997 (0.001)	(0.995, 0.999)	0.014*
PDGF-BB	1.005 (0.002)	(1.002, 1.008)	0.003**
EOTAXIN	0.977 (0.012)	(0.954, 1.000)	0.052
IL-16	0.999 (0.000)	(0.998, 0.999)	0.028*
SCF	1.0138 (0.009)	(0.997, 1.030)	0.107

^*^p-value <0.05, ^**^p-value <0.01, ^***^p-value <0.001.

**Table 5:** Multivariable Cox PH results for predictors of HIV survival among women aged 18–40 years (model 4).

**Variable**	**HR (SE)**	**95% CI**	***p*-value**
Other income source			
No	1.000		
Yes	2.807 (0.480)	(1.095, 7.197)	0.032*
Age of oldest sex partner	0.920 (0.033)	(0.862,0.982)	0.012*
Vaginal abnormal discharge			
No	1.000		
Yes	1.688 (0.318)	(0.906, 3.146)	0.099
Age at debut	0.824 (0.080)	(0.704, 0.963)	0.015*
Treatment group			
Placebo	1.000		
Tenoofovir	0.652 (0.185)	(0.454, 0.938)	0.021*
Sex in last 30 days	0.930 (0.035)	(0.868, 0.995)	0.037*
Salary			
No	1.000		
Yes	2.186 (0.454)	(0.899, 5.320)	0.085
Years lived in Durban	1.039 (0.019)	(1.001, 1.080)	0.045*
Site			
eThekwini			
Vulindlela	0.487 (0.411)	(0.218, 1.089)	0.080
SCF	1.040(0.009)	(1.022, 1.058)	0.000***
IL-15	0.909 (0.034)	(0.851, 0.971)	0.004**
MIP-1B	1.001 (0.000)	(1.000, 1.001)	0.001***
SCGF-B	0.994 (0.000)	(0.992, 0.999)	0.028*
GM-CSF	0.993 (0.003)	(0.988, 0.999)	0.018*
G-CSF	0.999 (0.000)	(0.999, 1.000)	0.098

^*^p-value <0.05, ^**^p-value <0.01, ^***^p-value <0.001.

The analysis result of model 1 in Table 2 indicates that age at enrolment was the only significant predictor of HIV hazard. Tenofovir treatment group reduced the hazard of HIV infection as compared to the Placebo treatment group (HR: 0.629, 95% CI: 0.405,0.977). The adjusted hazard ratio for a 1 year increase in age at enrolment is 0.949 (95% CI: 0.902, 0.998). This implies that HIV incidence decreases with increasing age.

Model 2 results in Table 3 shows that tenofovir treatment group reduced the hazard of HIV infection as compared to the placebo treatment group (HR: 0.486, 95% CI: 0.296, 0.798). For every average unit increase of the cytokines IL-12P70, IL-16, B-NGF, SCGF-B, IL-17A and IL-3 there is a decrease of HIV hazard by 2.32% (HR: 0.977, 95% CI: 0.968, 0.986), 0.44% (HR: 0.996, 95% CI: 0.999, 0.999), 19.3% (HR: 0.807, 95% CI: 0.670, 0.973), 0.07% (HR: 0.995, 95% CI: 0.993, 0.999), 2.14% (HR: 0.979. 95% CI: 0.965, 0.992) and 1.1% (HR: 0.989, 95% CI: 0.982, 0.996) respectively. On the other hand for every average unit increase of the cytokines SCF, TNF-A, CTACK, IL-10, IL-5 and IFN-A2 there is an increase of HIV hazard by 11.31% (HR: 1.113, 95% CI: 1.077, 1.151), 1.77% (HR: 1.018, 95% CI: 1.009, 1.027), 3.94% (HR: 1.039, 95% CI: 1.016, 1.063), 4.9% (HR: 1.049, 95% CI: 1.013, 1.086), 10.6% (HR: 1.106, 95% CI: 1.004, 1.220), and 2.75% (HR: 1.028, 95% CI: 1.006, 1.050) respectively.

After including the mean value of the cytokine measurements as covariate, the Cox model showed that age of the oldest sex partner, salary, years with stable partner and sex partner have other partner variables were significant baseline predictors associated with HIV infection. For every year increase for the age of the oldest sex partner, the hazard of HIV decreases by 5.47% (HR: 0.945, 95% CI: 0.898, 0.995). Patients who earned salary had a higher risk of HIV infection (HR: 2.474, 95% CI: 1.227, 4.987) compared to their counterparts who did not earn salary. It was surprising to note that, for every one additional stable partner there was about a 1.5 fold increase in hazard of HIV infection (HR: 1.480, 95% CI: 1.023, 2.138). Moreover, the patients whom did not know if their sex partners had other sex partners had a higher HIV hazard (HR: 2.948, 95% CI: 1.241, 7.001) than those who knew their sex partners did not have other sex partners. Testing the PH assumption using the Scaled Schoenfeld test for the significant variables indicated that CTACK (χ^2^: 4.710, df: 1, p: 0.03) did not meet the Cox PH assumptions.

The results of model 3 shown in Table 4 depicts that Tenofovir treatment group reduced the hazard of HIV infection as compared to the placebo treatment group (HR: 0.652, 95% CI: 0.238, 1.039). For every unit change (difference) of the cytokines B-NGF, IL-5, IL-16 and TRAIL there is a decrease of HIV infection by 10.38% (HR: 0.896, 95% CI: 0.851, 0.943), 7.7% (HR: 0.923, 95% CI: 0.882, 0.966), 0.08% (HR: 0.999, 95% CI: 0.998, 0.999) and 0.3% (HR: 0.997, 95% CI: 0.995, 0.999) respectively while for the same change in the cytokine CTACK, IL-2 and PDGF-BB there is an increase of HIV infection by 2.23% (HR: 1.022, 95% CI: 1.013, 1.032), 10.59% (HR: 1.106, 95% CI: 1.037, 1.179) and 0.49% (HR: 1.005, 95% CI: 1.002, 1.008) respectively. After including the difference value (between last observed and first value) of the cytokine measurements as covariate, the Cox model showed that sex partner have other partner, husband's income and age of the oldest sex partner covariate were significant baseline predictors associated with HIV infection. For every year increase of age for the oldest sex partner, HIV risk decreases by 5.47% (HR: 0.945, 95% CI: 0.898, 0.995). Both patients who did not know if their partners had other sex partners (HR: 2.948, 95% CI: 1.241, 7.001) and those who knew their sex partners had other partner (HR = 3.991, 95% CI: 1.535, 10.373) had a higher HIV hazard compared to those who knew their partners had no other sex partners. Additionally, the ones who received income from husband (HR: 1.901, 95% CI: 1.023, 3.534) had a higher hazard of HIV than those who did not receive any income from their husband. Testing the PH assumption using the Scaled Schoenfeld test indicated that all the significant variables from the model met the PH Cox assumptions.

Table 5 presents the analysis results of model 4 which indicates that tenofovir treatment group reduced the hazard of HIV infection as compared to the placebo treatment group (HR: 0.652, 95% CI: 0.454, 0.938). The cytokines IL-15, SCGF-B and GM-CSF had an instantaneous decrease of HIV incidence by 9.11% (HR: 0.909, 95% CI: 0.851, 0.971), 0.07% (HR: 0.994, 95% CI: 0.992, 0.999), and 0.68% (HR: 0.993, 95% CI: 0.988, 0.998) respectively at a particular time t. Conversely, SCF had an instantaneous increase of HIV incidence by 4.02% (HR: 1.040, 95% CI: 1.022, 1.058) at a particular time *t*. When using the cytokines as time dependent covariate, the Cox PH analysis indicated that significant baseline predictors were; age of oldest sex partner, other source of income, age at debut, sex in the last 30 days, and years lived in Durban. For every year increase of the age of the oldest sex partner and patient's age at debut, the hazard of HIV infection increases by 7.99% (HR: 0.920, 95% CI: 0.862, 0.982) and 17.63% (HR: 0.824, 95% CI: 0.704, 0.963) respectively. The less sex the patient had in the previous 30 days, the lower the patient's HIV risk by 7.04% (HR: 0.930, 95% CI: 0.868, 0.995). Furthermore, for every extra year the patient spends in Durban, the chance of HIV infection rises by 3.94% (HR: 1.039, 95% CI: 1.001,1.080). Likewise individuals with other sources of income had an increased risk of HIV infection by 180.68% (HR: 2.807, 95% CI: 1.095, 7.197) compared to those without. Upon testing the PH assumption on significant variables using scaled Schoenfeld residual test, other sources of income (χ^2^: 5.288, df: 1, *p*: 0.022) and age of oldest sex partner (χ^2^: 5.426, df: 1, *p*: 0.020) violated the PH assumption.

**Figure 4:**
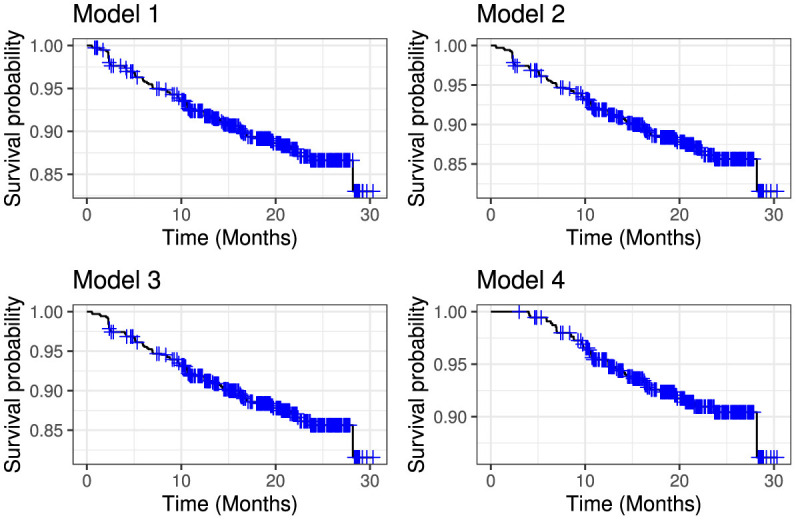
Comparative overall survival curves for model 1 (upper left panel), model 2 (upper right panel), model 3 (lower left panel) and model 4 (lower right panel).

The overall performance of the models (model 1–4) shown in Table 6 indicate that model 4 had the lowest AIC, while model 1 the highest AIC. The overall survival of the models over time are depicted in Figure 4.

**Table 6:** Comparative tests to evaluate Cox PH model performances.

	**Model 1**	**Model 2**	**Model 3**	**Model 4**
AIC (df)	1,064.5 (7)	919.3 (30)	955.3 (19)	531.4 (14)
Likelihood ratio test (df)	22.1 (7)	185.9 (30)	127.4 (19)	70.0 (14)
Wald test (df)	19.1 (7)	168.3 (30)	119.6 (19)	67.1 (14)
Logrank test (df)	19.9 (7)	248.9 (30)	136.9 (19)	102.4 (14)

**Figure 5:**
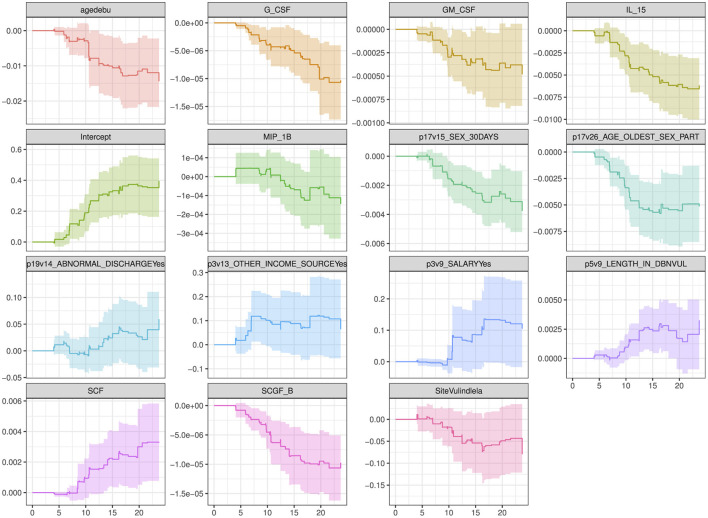
Overall trend of the covariate effects (only significant baseline and cytokine covariates) for model 4 over time. Abreviations of the baseline characteristics: agedebu, age at debut; p17v15_SEX_30DAYS, number of times had sex in 30 days; p17v26_AGE_OLDEST_SEX_PART, age of oldest sex partner; p19v14_ABNORMAL_DISCHARGEYes, abnormal discharge variable for yes category; p3v13_OTHER_INCOME_SOURCEYes, other income sources for category yes; p3v9_SALARYYes, salary variable for category yes; p5v9_LENGTH_IN_DBNVUL, number of years lived in Durban Vulindlela area; SiteVulindlela, site variable for category Vulindlela.

**Figure 6:**
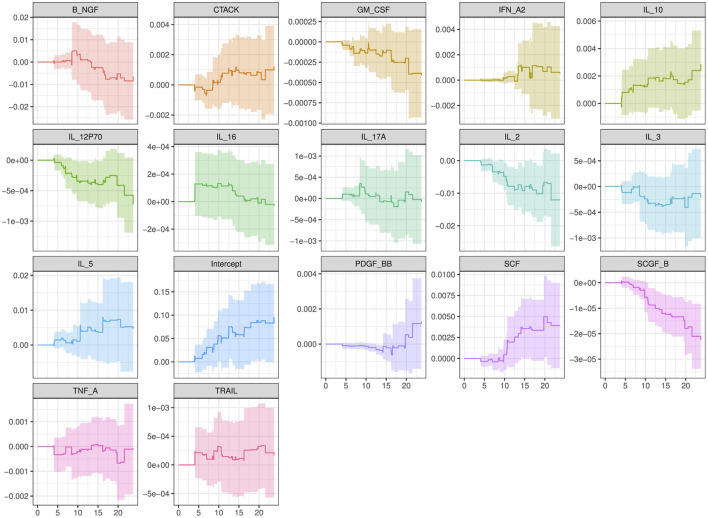
Direction of change for the cytokines of interest confirmed by our study results.

The plot in Figure 5 show how the effects of the covariates in model 4 (with the lowest model fit scores as shown in Table 6) change over time. The intercept of the model 4 in Figure 5 had a smooth increasing slope over time. The time dependent cytokine covariates; G-CSF, GM-CSF, IL-15, MIP-1B, SCGF-B, and baseline covariates; age at debut, sex in the last 30 days, age of oldest sex partner and site had a decreasing slope over time. Increasing slope over time is observed in the time-dependent covariate SCF and baseline covariates; abnormal discharge, other income source, salary and years lived in Durban. Table 7 indicate which cytokines overlap between model 2–4, or which are no longer significant in the subsequent models. Figure 6 illustrates the direction of change for the significant cytokines identified in the analysis of models 2–4.

**Table 7:** Significant predictors of HIV survival among women aged 18–40 years for model 2–4.

**Variable**	**Model 2**	**Model 3**	**Model 4**
IL-16	✓	✓	
MIF	✓	✓	
B-NGF	✓	✓	
CTACK	✓	✓	
IL-5	✓	✓	
MIG	✓		✓
SCGF-B	✓		✓
SCF	✓		✓
MIP-1B	✓		
MCP-1	✓		
IL-12P70	✓		
TNF-A	✓		
IL-17A	✓		
MCP-3	✓		
IL-10	✓		
G-CSF	✓		
IL-3	✓		
IL-2RA	✓		
IFN-A2	✓		
IL-1RA		✓	
IL-2		✓	
TRAIL		✓	
PDGF-BB		✓	
IL-15			✓
GM-CSF			✓

The table indicate which cytokines overlap between the different models, or which are no longer significant in the subsequent models.

Blank: cytokine absent in the model.

✓: cytokine was significant in the model.

## 4 Discussion

The global HIV pandemic remains a significant public health challenge, necessitating the continuous exploration of innovative preventive strategies (45). Pre-exposure prophylaxis (PrEP) particularly Antiretroviral Microbicide has emerged as a promising intervention for individuals at high risk of HIV acquisition (4). However, variations in individual immune responses, particularly in cytokine profiles, may influence the efficacy of PrEP. It is known that dynamic changes in immune states are linked with HIV acquisition, and biomarkers, demographic and behavioral data add complementary details to HIV risk (21). Recent research has highlighted the potential of cytokines as biomarkers in the Pre-exposure prophylaxis. Cytokines have been suggested as potential predictors of HIV acquisition.

This study investigated the effect of individual cytokine biomarkers that changes over time in determining HIV incidence among individuals randomized to PrEP vs. control exposure by building a series of Cox Proportional Hazard models. The Cox PH is essentially a regression model commonly used statistical method in medical research and in other applications for investigating the association between the survival time and one or more predictor variables (16). The simple form of Cox model is when it models time fixed covariates. One of the strengths of the extended Cox model is its ability to incorporate covariates that change over time. This functionality is practical because, at each event time, the Cox model compares the current covariate values of the subject experiencing the event with the current values of all other subjects who were at risk at that time (41). We incorporate stepwise regression in the Cox PH model to eliminate noisy variables and remain with the best model fit (31).

The cytokine biomarkers in our data set changes over time i.e they were longitudinally measured, indicating the presence of a time-varying covariates. When such covariates exist, an analyst should consider taking them into account in survival modeling in order to improve estimation (15). The presence of time-dependent covariates in a model offers exciting opportunities for exploring associations and potentially causal mechanism (46). However, the use of these variables is technically difficult in the choice of covariate form, might have great potential for bias and violates the assumption that the hazard ratio for any two individual remains constant over time. We therefore, improve the model fit by using derived cytokine variables from the longitudinal measurements. As a starting point in modeling, we started with Model 1 (Equation 8), a traditional time-invariant (baseline covariates) Cox PH model. In this model the initial variables were 24 which were further reduced to seven variables that contributed to the best model fit and it estimated age at enrollment as the only significant predictors of HIV risk.

The first improved model (model 2-Equation 9) we used baseline covariates plus the individual level average of the cytokine measurements to better describe the average effect of the time-varying cytokine covariate. Through stepwise regression the covariates were reduced from 72 to 30 in the final best fit model. When comparing model 1 with model 2 we are able to identify four other different baseline covariate (Age of oldest sex partner, salary, years with stable partner and sex partner having other sex partner) and twelve individual average cytokines (IL-3, IL-5, IL-10, IL-16, IL-17A, IL-12P70, CTACK, SCF, B-NGF, SCGF-B, TNF-A, IFN-A2) that are significantly associated with HIV risk. Therefore, the predictive performance of model 2 was better than model 1 with lower AIC (919.3) in comparison to model 1 AIC (1,064.5). This clearly showed that not accounting for cytokine effect in model 1 confounded the effect of other significant baseline characteristics.

Model 3 (Equation 10) is the second improved Cox PH model which consisted of baseline covariates plus individual cytokine difference between the first and the last observed measurement. The final best model fit in model 3 had 19 covariates from an initial total of 72. Notably the model had a better predictive performance compared to model 1 as it had three additional baseline covariates (sex partner having other partners, husband's income, age of the oldest sex partner) and seven individual difference cytokines (IL-2, IL-5, IL-16, CTACK, PDGF-BB, BNG-F and TRAIL) that were significant predictors of HIV infection. Furthermore, when compared to model 2, there were three similar baseline covariates (age of the oldest sex partner, treatment group and sex partner having other partner) that were significant predictors in both models. However, there were fewer cytokine covariates than in model 2, with IL-5, IL-16, CTACK, and BNG-F all being significant cytokine covariates in both models. When compairing the AIC of the models, model 3 had a lower AIC than model 1 but slightly higher than AIC of model 2. Model 3 predicted the individual level changes of the cytokines and its association with HIV risk therefore accounting for time. The major drawback of the model was some individuals had single measurements hence no change effect observed. Additionally, the model ignores the intermediate changes between the first and the last observed cytokine measurement which implies loss of information within individual cytokine measurements.

The last improved Cox PH model fit was model 4 (Equation 11) that used baseline covariates plus time-dependent cytokine covariates. The final best model fit consisted of 14 variables out of 72. When compared to model 1, there were five additional baseline covariates (age of oldest sex partner, age at debut, other income source, sex in the last 30 days and years lived in Durban) and four time-dependent cytokines (SCF, IL-5, SCGF-B and GM-CSF). Moreover, Age of oldest sex partner and IL-5 were significant predictors estimated by all the improved models while SCF and SCGF-B were both predictors by model 2 and 4. Likewise CTACK, IL-5, IL-16 and B-NGF were significant predictors estimated in both model 3 and 4. Table 7 indicate which cytokines overlap between models 2–4, or which are no longer significant in the subsequent models.

Overall, model 2 produced the greatest number of significant cytokine predictor variables, giving a wider perspective to a researcher which cytokine biomarkers are associated with HIV Hazard. However, there is loss of time information in this model for the derived cytokine variables. Model 4 on the other hand had the lowest AIC compared to the other models making it the best model. This emphasizes that time-dependent covariates is a powerful tool for exploring predictive relationships. Nevertheless, their use and interpretation is much more complicated in practice than the fixed (baseline) covariates. Furthermore the potential for erroneous inference and modeling is increased (46).

Our findings reveal that incorporating cytokine biomarkers into the PH regression model not only enhances the model's predictive performance but also provides more insightful information about significant predictors linked to HIV incidence. These results are consistent with a recent study by Ignacio et al. (21), which found that changes in cytokine levels over time are highly predictive of HIV acquisition and that cytokines influence the effects of sociobehavioral risk factors on HIV acquisition. Although Ignacio et al. (21) used a different model (LASSO machine learning algorithms), a different dataset (Sabes study), and selected fewer biomarkers (10 cytokines), their study also highlighted the importance of immune activation markers in predicting HIV beyond traditional demographic and behavioral factors, aligning with our objective. Our analysis identified and reported several baseline predictors such as the age of the oldest sex partner, participant's age at enrollment, earning a salary or not, years with a stable partner, income source, whether the sex partner has other partners, and frequency of sex in the last 30 days as significantly associated with HIV incidence. These findings align with those of other research studies (47–55).

In the previous analysis by Masson et al. (18) to investigate whether genital inflammation influenced HIV acquisition in women, they used 12 cytokines out of 48 available cytokine measurements. This selection was disadvantageous as it excluded other potentially relevant cytokine covariates. They utilized conditional logistic regression which has limitations because the risk sets and time-dependent covariates are predefined, unlike in Cox regression, where these factors are calculated at the time of each case failure (56). Moreover, Cox models that was employed in our study, offers more statistical power than logistic regression models because they account for the time until the event occurs (57). Naranbhai et al. using the same dataset, employed logistic regression and PCA to investigate the role of immune activation in HIV acquisition. The PCA's assumption of linearity limits its effectiveness in interpreting the components, as they are linear combinations of the original variables (58). Ngcobo et al. (20), in their study of examining whether pre-infection plasma cytokine concentrations predicted the rate of HIV disease progression in the same study cohort, used linear regression to assess the impact of each cytokine on viral load (VL) and the CD4:CD8 ratio in both bivariate and multivariable models. The major drawback of linear regression is its lack of consideration for time continuity (56). Notably, none of the previous studies exploring predictors of HIV progression (20, 22, 23) using CAPRISA 004 trial considered cytokine biomarkers as time-varying covariates. This study underscores the importance of incorporating longitudinal risk factor information in predicting HIV incidence.

Our study results successfully confirmed the cytokines Interleukin (IL-2, IL-3, IL-5, IL-10, IL-16, IL-12P70, and IL-17 alpha), Stem cell factor (SCF), Beta Nerve growth factor (B-NGF), Tumor necrosis factor alpha (TNF-A), interferon (IFN) alpha-2, serum stem cell growth factor (SCG)- beta, platelet-derived growth factor (PDGF)-BB, Granulocyte macrophage colony stimulating factor (GM-CSF), tumor necrosis factor-related apoptosis-inducing ligand (TRAIL) and cutaneous T-cell-attracting chemokine (CTACK) are associated directly to HIV infection and identified new cytokine biomarkers to enrich the field's literature further. Figure 6 shows the direction of change for the cytokines mentioned. Therefore, better understanding of the role of cytokines before, during, and after HIV infection could enable for the development of new therapeutic approaches based on the use of either recombinant cytokines or particular antagonists, with the goal of limiting both HIV spread and clinical manifestations of this infection (59).

Different cytokines play significant roles in HIV prevention and management with PrEP. Interleukins (ILs) such as IL-2 enhance T-cell proliferation and activation, aiding the immune response against HIV, and its levels can help assess immune activation efficacy in PrEP users. IL-3 and IL-5 regulate hematopoiesis and immune responses, with elevated levels indicating an ongoing immune response relevant for those exposed to HIV (60). IL-10, an anti-inflammatory cytokine, prevents excessive inflammation, with high levels suggesting reduced inflammation in PrEP patients. IL-12P70 and IL-17 alpha aid in differentiating and activating T-helper cells, promoting cell-mediated immunity, and protecting mucosal barriers, respectively (60). Monitoring these cytokines helps understand immune modulation in PrEP users. IL-16 attracts T-cells and other immune cells to infection or inflammation sites, a marker for immune activation in PrEP.

SCF and SCG-beta are essential for hematopoietic stem cell proliferation and differentiation, indicating bone marrow activity and the ability to replenish immune cells in PrEP users (61). B-NGF supports neuron survival and maintenance and has immunomodulatory effects. In PrEP users, B-NGF might influence neuroimmune interactions, affecting the nervous system's response to HIV exposure. TNF-A and TRAIL are significant in immune regulation and inflammation (10). TNF-A, a pro-inflammatory cytokine, indicates inflammation and immune activation, which is crucial for those at risk of HIV. TRAIL induces apoptosis in cancer and infected cells, helping eliminate HIV-infected cells in PrEP users. IFN Alpha-2 has antiviral properties, inhibiting HIV replication and modulating the immune response, providing additional protection in PrEP users. PDGF-BB aids in wound healing and tissue repair, helping maintain mucosal integrity and prevent HIV entry through mucosal surfaces in PrEP users. GM-CSF stimulates granulocyte and macrophage production, providing insights into immune readiness (62). CTACK directs T-cells to the skin, indicating immune surveillance at mucosal and skin surfaces to prevent initial HIV infection.

In clinical practice, these cytokines are useful biomarkers to monitor individuals' immune status and response using PrEP. Regularly measuring cytokines like IL-2, IL-10, TNF-A, IFN Alpha-2, IL-12P70, IL-17 alpha, and TRAIL can help assess immune activation, regulation, and the body's response to HIV exposure (60). This monitoring allows clinicians to evaluate the balance between immune activation and regulation, ensuring optimal immune response without excessive inflammation (11). Personalized PrEP strategies can be developed based on individual cytokine profiles, optimizing dosages and combinations of PrEP medications to enhance protection. Additionally, certain cytokines can indicate adverse immune reactions or inflammation, enabling timely interventions to manage side effects. Integrating cytokine monitoring into PrEP care enhances HIV prevention strategies, tailored interventions to individual needs, and improves clinical outcomes.

## 5 Conclusion

In this article we investigated the effect of individual cytokine biomarker, a time varying covariate, where we provided ways of handling the covariate in the stepwise Cox PH modeling by using a derived variable from the longitudinal measurements (mean and difference) and as a time dependent covariate (model 2–4). The presence of a cytokine effect in a model improved the predictive performance of the model hence the improved models were more informative about predictors that are associated with HIV hazard. Moreover, the tenofovir treatment exposure significantly lowered the hazard of HIV compared to the Placebo treatment group. Furthermore, Kaplan–Meier estimator indicated that the patients who received tenofovir antiretroviral microbicide treatment had a significantly lower risk of HIV infection compared to the placebo group hence an effective treatment in reducing the risk of HIV in women between the age of 18–40 years.

Further investigation of the cytokine biomarker could involve utilizing the standard deviation of longitudinal measurements or lagged observations. Additionally, with internal time-varying covariates, one might explore employing joint modeling of longitudinal and survival data. The aim is to apply a model to a continually changing covariate that is measured longitudinally, potentially with error. This longitudinal model is linked to survival times by modeling the joint distribution of longitudinal and survival data.

## Data availability statement

The data analyzed in this study is subject to the following licenses/restrictions. Researchers wanting to access data from the completed CAPRISA studies are requested to complete a data request form. Requests to access these datasets should be directed to https://www.caprisa.org/Pages/CAPRISAStudies.

## Author contributions

SO: Writing – review & editing, Writing – original draft, Visualization, Validation, Software, Resources, Project administration, Methodology, Investigation, Formal analysis, Data curation, Conceptualization. MM: Writing – review & editing, Visualization, Validation, Supervision, Software, Formal analysis. HM: Writing – review & editing, Validation, Supervision, Methodology, Conceptualization.
