# Supplementary material for: Investigating the effects of cytokine biomarkers on HIV incidence: a case study for individuals randomized to pre-exposure prophylaxis vs. control
Source: Front Public Health. 2024 Jun 25;12:1393627. doi: 10.3389/fpubh.2024.1393627 (PMC11231092; doi:10.3389/fpubh.2024.1393627)
Supplement: Supplementary file 1 [file Data_Sheet_1.PDF]

Supplementary Material

1 SUPPLEMENTARY TABLES

Table S1. Variable description

Variable name	Description
Treat	Randomization arm (Placebo, Tenofovir)
Site	Clinical location (eThekwini, Vulindlela)
Months	Months of exposure in the study
HIV	HIV indicator variable (1=Positive; 0=Negative)
p2v18_REG_PARTNER_LIVE_TOGETHER	Patient and regular partner live together (No, Yes)
p2v22_HIGHEST_EDUCATION	Highest Level Of Education (High School, Primary,tertiary)
p3v8_SELF_GEN_INCOME	Income Source - Self Generated Income (No, Yes)
p3v9_SALARY	Income Source - Salary/Wage (No, Yes)
p3v10_HUSBAND	Husband Partner (No, Yes)
p3v11_SOCIAL_GRANTS	Income Source - Social Grants/Remits (No, YEs)
p3v12_NO_INCOME	Income Source - No Income
p3v13_OTHER_INCOME_SOURCE	Income Source - Other (No, Yes)
p3v16_AMOUNT_INCOME	Amount Income (¡R1000 Per Month, R1001-R5000 Per Month)
p5v9_LENGTH_IN_DBNVUL	Years lived in Durban/Vulindlela Area
p6v8_TIMES_HAD_VAG_SEX	Times Had Sex In Last 30 Days
age1	Age at enrollment
marital	Marital status (Casual, Married, Stable, Stable & Casual)
agedebu	Age At Sexual Debut
contra2	Contraceptives
p17v10_PARTNERS	Total Number Of Sexual Partners
p17v11_YEAR_STABLE	Num Stable Sex Partners In Past Year
p17v12_YEAR_CASUAL	Num Casual Sex Partners In Past Year
p17v13_30DAYS_STABLE	Num Stable Sex Partners In 30 Days
p17v14_30DAYS_CASUAL	Num Casual Sex Partners In 30 Days
p17v15_SEX_30DAYS	Times Had Sex In Last 30 Days
p17v17_VAG	Type Of Sex Acts - Vag Only
p17v18_ANAL	Type Of Sex Acts - Anal Only
p17v19_ORAL	Type Of Sex Acts - Oral Only
p17v20_VAG_ANAL	Type Of Sex Acts - Vag And Anal
p17v21_VAG_ORAL	Type Of Sex Acts - Vag And Oral
p17v25_ANY_NEW_PARTNERS	Any New Sex Partners In Last 30 Days
p17v26_AGE_OLDEST_SEX_PART	Oldest Sex Partner Age In Last 30 Day
p17v28_SEX_PART_HAVE_OTHER	Sex Partners Have Other Sex Partners
p18v8_SEX_PART_TEST_POS	Any Sex Part In Last 30 Day Test HIV+ (Don't know, No, Yes)
p18v11_RECEIVED_MONEY	Ever Received Money/Gifts For Sex
p18v12_RECEIVE_MONEY_30DAYS	Receive Money Last 30 Days For Sex
p18v13_FREQ_ALCOHOL	Frequency Of Alcohol Consumption
p18v15_FREQ_CONDOM_USE	Frequency Of Condom Use (Always, Occassionally)
p18v16_FREQ_CONDOM_STABLE	Freq Of Condom Use By Stable Partner (Always,Mostly, Never, Occassionally)
p18v17_FREQ_CONDOM_CASUAL	Freq Of Condom Use By Casual Part
p19v14_ABNORMAL_DISCHARGE	Abnormal Genital/Vaginal Discharge (No, Yes)
HSV2	HSV-2 results
p19v8_ITCHING	Genital/Vaginal Itching (No, Yes)
p19v11_PAIN_DURING_SEX	Pain During Sex
p43v9_TIMES_SEX	Number of times had sex
p43v15_NUM_STABLE_PARTNERS	Number of Stable Sexual Partners
p43v16_NUM_CASUAL_PARTNERS	Number of Casual Sexual Partners
p43v17_NUM_NEW_PARTNERS	Number of New Partners
p43v21_OTHER_SEXUAL_PARNERS	Sexual Partners Other Than Your Self (Yes, No)
p44v9_BURNING	Genital/Vaginal Burning (No, Yes)
p44v10_PAIN	Genital/Vaginal Pain (No, Yes)
p44v12_DIFFICULTY_URINATING	Difficult/Burning When Urinating (No, Yes)
p44v13_VAGINAL_BLEEDING_SPOTTING	Vaginal Bleeding Or Spotting (No, Yes)
p44v15_OTHER_GENITO_ULCERS	Other Genitourinary Ulcers (No, Yes)

Table S2. Frequency distribution for baseline categorical variables for negative HIV women aged 18-40 years (N=755)

Variable	Frequency	Percentage
**Treat**		
**Placebo**	374	49.54
**Tenofovir**	381	50.46
**Site**		
**eThekwini**	247	32.72
**Vulindlela**	508	67.28
**Partner live together**		
**No**	654	86.62
**Yes**	101	13.38
**Highest level of Education**		
**High School**	666	88.21
**Primary**	37	4.90
**Tertiary**	52	6.89
**Self generated Income**		
**No**	721	95.50
**Yes**	34	4.50
**Salary**		
**No**	671	88.87
**Yes**	84	11.13
**Husband's Income**		
**No**	654	86.62
**Yes**	101	13.38
**Social Grants**		
**No**	151	20.00
**Yes**	604	80.00
**Other Income Source**		
**No**	689	91.23
**Yes**	66	8.77
**Amount of Income**		
**R1001-R5000 per month**	73	9.67
**¡R1000 per month**	682	90.33
**Marital Status**		
**Casual**	10	1.32
**Married**	47	6.23
**Stable & Casual**	32	4.24
**Stable**	666	88.21
**Sex partner have other partner**		
**Don't Know**	483	63.97
**No**	115	15.23
**Yes**	157	20.80
**Vaginal abnormal discharge**		
**No**	514	68.08
**Yes**	241	31.92
