# Supplementary material for: Investigating the effects of cytokine biomarkers on HIV incidence: a case study for individuals randomized to pre-exposure prophylaxis vs. control
Source: Front Public Health. 2024 Jun 25;12:1393627. doi: 10.3389/fpubh.2024.1393627 (PMC11231092; doi:10.3389/fpubh.2024.1393627)
Supplement: Supplementary file 2 [file Data_Sheet_2.pdf]

Statistical Analysis Code

Sarah Ogutu

2024-06-03

R packages

```
suppressPackageStartupMessages({
  library(readxl)
  library(fpp3)
  library(tidyverse)
  library(dplyr)
  library(survival)
  library(ranger)
  library(ggplot2)
  library(ggfortify)
  library(mice)
  library(finalfit)
  library(VIM)
  library(gtsummary)
  library(survminer)
  library(MASS)
  library(StepReg)
  library(kableExtra)
  library(tableone)
  library(brolgar)
  library(xtable)
  library(gghighlight)
  library(patchwork)
  library(tableone)
  library(xtable)
  library(GGally)
  library(eha)
  library(flexrsurv)
  library(caret)
})
```

Data Set

```
HIV1 <-read_excel("G:/My Drive/PhD STAT/Survival Analysis/HIV1.xlsx")
HIV1 <-as.data.frame(unclass(HIV1),stringsAsFactors=TRUE)
Base <- read_excel("G:/My Drive/PhD STAT/Survival Analysis/Base.xlsx",
                   na=c(".", "Blank", "D", "A"))
Cytokines <- read_excel("G:/My Drive/PhD STAT/Survival Analysis/Cytokines.xlsx")
```

Data pre-processing

```
# Check data structure
str(Base) # baseline covariates
str(HIV1)
str(Cytokines) #Cytokine Profile

# Convert categorical variables into factor variables
Base_modified = Base
Base_modified <- as.data.frame(unclass(Base_modified),stringsAsFactors=TRUE)
Base_modified$agedebu <-as.numeric(Base_modified$agedebu)

# Display levels and frequencies of factor variables
lapply(Base_modified[sapply(Base_modified, is.factor)], table)

# merge data set
Surv_list2<- list(HIV1, Base_modified)
Base_surv_analysis<- Surv_list2 %>% reduce(inner_join, by='PID')

# renaming the string of factor variables
Base_surv_analysis1 <- Base_surv_analysis %>%
  mutate(p2v18_REG_PARTNER_LIVE_TOGETHER = str_replace(
    p2v18_REG_PARTNER_LIVE_TOGETHER, "No Regular Partner", "No"))

Base_surv_analysis2 <-Base_surv_analysis1 %>%
  mutate(p2v22_HIGHEST_EDUCATION = str_replace_all(
    p2v22_HIGHEST_EDUCATION, c("High School Complete"="HSchool",
                               "High School Not Complete"="HSchool",
                               "Tertiary Education Complete"= "Tertiary",
                               "Tertiary Education Not Complete"="Tertiary",
                               "Primary Schooling Complete"="Primary",
                               "Primary School Not Complete"="Primary",
                               "No Schooling" ="Primary")))

Base_surv_analysis3 <- Base_surv_analysis2 %>%
  mutate(p3v8_SELF_GEN_INCOME = str_replace_all
         (p3v8_SELF_GEN_INCOME,
           c("No self Gen Income"="NoSelf", "Self Gen Income"="Self")))
Base_surv_analysis3$
  p3v8_SELF_GEN_INCOME[Base_surv_analysis3$p3v8_SELF_GEN_INCOME==
                         "No Self"] <- "No"
Base_surv_analysis3$
  p3v8_SELF_GEN_INCOME[Base_surv_analysis3$p3v8_SELF_GEN_INCOME==
                         "Self"] <- "Yes"

Base_surv_analysis4<-Base_surv_analysis3 %>%
  mutate(p3v9_SALARY =
           str_replace_all(p3v9_SALARY,
                           c("Salary/Wage"="Yes","No Yes"="No")))
Base_surv_analysis4$
  p3v9_SALARY[Base_surv_analysis4$p3v9_SALARY=="No Yes"] <- "No"

Base_surv_analysis5<-Base_surv_analysis4 %>%
```

```
  mutate(p3v10_HUSBAND =
           str_replace_all(p3v10_HUSBAND, c(
    "Income Husband/Stable Partner"="Yes",
    "Not Husband/Stable Partner"="No")))

Base_surv_analysis6<-Base_surv_analysis5 %>%
  mutate(p3v11_SOCIAL_GRANTS = str_replace_all(
    p3v11_SOCIAL_GRANTS, c("No Social Grant"="No", "Social Grant"="Yes")))
Base_surv_analysis6$
  p3v11_SOCIAL_GRANTS[Base_surv_analysis6$p3v11_SOCIAL_GRANTS=="Nos"] <- "No"
Base_surv_analysis6$
  p3v11_SOCIAL_GRANTS[Base_surv_analysis6$p3v11_SOCIAL_GRANTS=="Yess"] <- "Yes"

Base_surv_analysis7<-Base_surv_analysis6 %>%
  mutate(p3v12_NO_INCOME =
           str_replace_all(p3v12_NO_INCOME,
                           c("Income"="No", "No Income"="Yes")))

Base_surv_analysis7$p3v12_NO_INCOME[Base_surv_analysis7$
                                      p3v12_NO_INCOME=="No No"] <- "Yes"

Base_surv_analysis8<-Base_surv_analysis7 %>%
  mutate(p3v13_OTHER_INCOME_SOURCE =
           str_replace_all(p3v13_OTHER_INCOME_SOURCE,
                           c("No Other Income Source"="No",
                             "Other Income Source"="Yes")))

Base_surv_analysis9<-Base_surv_analysis8 %>%
  mutate(p3v16_AMOUNT_INCOME =
           str_replace_all(p3v16_AMOUNT_INCOME,
                           c("<R1000 Per Month"="less1K",
                             "R1001-R5000 Per Month"="G1Kless5K")))

Base_surv_analysis10 <- Base_surv_analysis9 %>%
  mutate(marital = str_replace(marital, "Stable and Casual", "StabCas"))
Base_surv_analysis10$marital[Base_surv_analysis10$marital==
                               "Stable and Casual"] <- "StabCas"
Base_surv_analysis10$marital[Base_surv_analysis10$marital==
                               "Married and Casual"] <- "Married"

Base_surv_analysis11 <- Base_surv_analysis10 %>%
  mutate(p17v28_SEX_PART_HAVE_OTHER =
           str_replace(p17v28_SEX_PART_HAVE_OTHER,
             "Don't Know", "DontKn"))

Base_surv_analysis12 <- Base_surv_analysis11 %>%
  mutate(p18v8_SEX_PART_TEST_POS =
           str_replace(p18v8_SEX_PART_TEST_POS,"Don't Know", "DontKn"))
Base_surv_analysis13 <- Base_surv_analysis12 %>%
  mutate(p18v13_FREQ_ALCOHOL =
           str_replace(p18v13_FREQ_ALCOHOL,"Most Times", "Mostly"))
Base_surv_analysis14 <- Base_surv_analysis13 %>%
  mutate(p18v15_FREQ_CONDOM_USE =
```

```r
           str_replace(p18v15_FREQ_CONDOM_USE, "Most Times", "Mostly"))
Base_surv_analysis15 <- Base_surv_analysis14 %>%
  mutate(p18v16_FREQ_CONDOM_STABLE =
           str_replace(p18v16_FREQ_CONDOM_STABLE,"Most Times", "Mostly"))
Base_surv_analysis16 <- Base_surv_analysis15 %>%
  mutate(p18v17_FREQ_CONDOM_CASUAL =
           str_replace(p18v17_FREQ_CONDOM_CASUAL,"Most Times", "Mostly"))
Base_surv_analysis16 <- Base_surv_analysis16 %>%
  mutate(HSV2 = str_replace(HSV2, "assumed negative at baseline", "Negative"))

Base_surv_analysis16 <- Base_surv_analysis16 %>%
  mutate(contra2 = str_replace(contra2, "Hyst", "Tuba"))

Base_surv_analysis16$
  p17v28_SEX_PART_HAVE_OTHER[Base_surv_analysis16$
                             p17v28_SEX_PART_HAVE_OTHER==
                             "Don't know"] <- "Dontknow"
Base_surv_analysis16$p18v8_SEX_PART_TEST_POS[Base_surv_analysis16$
                                               p18v8_SEX_PART_TEST_POS==
                                               "Don't know"] <- "Dontknow"

Base_surv_analysis16 <- as.data.frame(unclass(Base_surv_analysis16)
                                      ,stringsAsFactors=TRUE)

# Check missingness
sum(is.na(Base_surv_analysis16)) # total missing values

Base_surv_analysis16 %>%summarise_all(~sum(is.na(.))) #total missing in column

# removing  high missingness, fewer levels and frequencies
Base_surv_analysis16.1 <- subset(Base_surv_analysis16,
                             select = -c(p18v17_FREQ_CONDOM_CASUAL,
                                         p17v18_ANAL, contra2, p17v17_VAG,
                                         p17v19_ORAL,p17v20_VAG_ANAL,
                                         p17v21_VAG_ORAL,
                                         p17v25_ANY_NEW_PARTNERS,
                                         p18v11_RECEIVED_MONEY,
                                         p18v11_RECEIVED_MONEY,
                                         p18v12_RECEIVE_MONEY_30DAYS,
                                         p18v13_FREQ_ALCOHOL,
                                         HSV2, p19v9_BURNING,
                                         p19v10_PAIN, p19v11_PAIN_DURING_SEX,
                                         p19v12_DIFF_URINATING,
                                         p19v13_VAG_BLEEDING,
                                         p19v15_OTHER_ULCERS, p3v12_NO_INCOME,
                                         p18v16_FREQ_CONDOM_STABLE,
                                         p18v8_SEX_PART_TEST_POS,
                                         p19v8_ITCHING,
                                         p6v8_TIMES_HAD_VAG_SEX))
Base_surv_analysis16.1<- Base_surv_analysis16.1 %>% na.omit()

# Releveling the factor variables
Base_surv_analysis16.1 <- within(Base_surv_analysis16.1,
```

```
                                  p2v18_REG_PARTNER_LIVE_TOGETHER<-
                                    relevel(p2v18_REG_PARTNER_LIVE_TOGETHER,
                                            ref = "Yes"))
Base_surv_analysis16.1 <- within(Base_surv_analysis16.1,
                                  p17v28_SEX_PART_HAVE_OTHER<-
                                    relevel(p17v28_SEX_PART_HAVE_OTHER,
                                            ref = "No"))

Base_surv_analysis16.1 <- within(Base_surv_analysis16.1,
                                  p2v18_REG_PARTNER_LIVE_TOGETHER<-
                                    relevel(p2v18_REG_PARTNER_LIVE_TOGETHER,
                                            ref = "Yes"))

# checking the total ID for Cytokine data set
n_distinct(Cytokines$PID)

# adjusting the visit codes to follow up months
Cytokines1 = mutate(Cytokines,
                    DFSEQ = case_when(DFSEQ == 1010~1, DFSEQ == 1020~2,
                                      DFSEQ == 1021~2.1, DFSEQ==1030~3,
                                      DFSEQ == 1031~3.1, DFSEQ == 1040~4,
                                      DFSEQ == 1050~5, DFSEQ == 1060~6,
                                      DFSEQ == 1070~7, DFSEQ == 1080~8,
                                      DFSEQ== 1090~9, DFSEQ == 1100~10,
                                      DFSEQ == 1110~11, DFSEQ == 1120~12,
                                      DFSEQ== 1130~13, DFSEQ == 1140~14,
                                      DFSEQ == 1141~14.1, DFSEQ==1150~15,
                                      DFSEQ == 1160~16, DFSEQ==1170~17,
                                      DFSEQ == 1180~18, DFSEQ==1190~19,
                                      DFSEQ == 1200~20, DFSEQ==1210~21,
                                      DFSEQ == 1220~22, DFSEQ==1230~23,
                                      DFSEQ == 1240~24, DFSEQ==1250~25,
                                      DFSEQ == 1260~26, DFSEQ==1270~27,
                                      DFSEQ == 1280~28, DFSEQ==1290~29,
                                      DFSEQ == 5999~30, DFSEQ==6000~31)
)
Cytokines1 <- Cytokines1 %>% rename("Follow_up_Month" = "DFSEQ")
```

Exploratory analysis: Graphs and tables

```
# Plot for number of cytokine measurement
Cytokine_A<-as_tsibble(Cytokines,
                      key = PID,
                      index = DFSEQ,
                      regular = FALSE,
                     validate = FALSE)
# Figure 1
Figure_1 <- Cytokine_A %>%
  features(BASIC_FGF, n_obs) %>%
  ggplot(aes(x = n_obs)) +
  geom_bar()+labs(x="Number of cytokine measurements", y="Count")+
  geom_text(stat='Count', aes(label=..count..), vjust=-0)+theme_bw()
```

```
# Figure 2
# Proportion and patterns for missing values in Baseline covariates
Base_surv_analysis16_A <-subset(Base_surv_analysis16, select = -c(p18v17_FREQ_CONDOM_CASUAL))
aggr_plot <- aggr(Base_surv_analysis16_A, col=c('navyblue','yellow'),
                  numbers=TRUE, sortVars=TRUE,
                  labels=names(Base_surv_analysis16_A), cex.axis= 0.4,
                  gap=2.5, cex.numbers = 0.7, cex.lab = 1, prop = TRUE,
                  ylab=c("Proportion of missing data",
                         "Pattern of missing data"))

# table one
factorVars <- c(names(Filter(is.factor,Base_surv_analysis16.1))) #categorical variables
vars <- dput(names(Base_surv_analysis16.1))
tableOne <- CreateTableOne(vars = vars, strata =
                             c("HIV", "Treat"),
                           data = Base_surv_analysis16.1,
                           factorVars = factorVars) # stratified by trt
tableOne
tableOne$CatTable
tableOne$ContTable
tabAsStringMatrix <- print(tableOne, printToggle = FALSE, noSpaces = TRUE)
xtable(tabAsStringMatrix) # read to latex
```

Kaplan-Meier Analysis

```
# Survival table
km_fitt <- survfit(Surv(months,HIV) ~ 1, data=HIV1)
km_table <-summary(km_fitt, times = c(1:30))

km_table1 <- cbind(km_table$time, km_table$n.risk, km_table$n.event,
                   km_table$n.censor, km_table$surv, km_table$std.err,
                   km_table$lower, km_table$upper)

km_table1 %>%as.data.frame() %>%
  kbl(caption = "Example1", format = "latex", digits = 4) #Importing to latex

#overall survival plot
km1 <-autoplot(km_fitt)+labs(x="Months", y="Survival probabilty")+theme_bw()

# Figure 3
# By treatment survival plot
km_fitt_trt <- survfit(Surv(months,HIV) ~ Treat, data=HIV1)
km2<- autoplot(km_fitt_trt)+
  labs(x="Months", y="Survival probability",
       colour = "Treat", fill = "Treat")+theme_bw() +
  annotate("text", x = 7, y = 0.8,
                   label = "Chisq = 5.7, \n df = 1, \n p = 0.02", size = 3)

km_plot <- km1+km2
ggsave("Figure3.pdf", plot = km_plot, width = 8, height = 5,
       units = "in", dpi = 600, device = "pdf")
```

```r
# log rank for kaplan meiyer when compairing two groups
surv_diff <- survdiff(Surv(months,HIV) ~ Treat, data=HIV1)
surv_diff
```

Stepwise Cox PH Analysis

Model 1

```r
# the Cox formulae
form1 = Surv(months, HIV) ~ .
# Stepwise Cox regression
Base_surv_analysis16.2 <- subset(Base_surv_analysis16.1,
                             select = -c(PID))
Base_results <- stepwiseCox(formula=form1,
                          data =  Base_surv_analysis16.2,
                          selection = "bidirection",
                          select = "AIC",
                          sle = 0.15,
                          sls = 0.15,
                          method = "efron")

# Using the selected variables from the stepwise regression
vec_base <- as.vector(rownames(Base_results$`Coefficients of the Selected Variables`))
vec_base[vec_base=="Treat2"] <- "Treat"
vec_base[vec_base=="p19v14_ABNORMAL_DISCHARGE2"] <- "p19v14_ABNORMAL_DISCHARGE"
vec_base[vec_base=="p3v8_SELF_GEN_INCOME2"] <- "p3v8_SELF_GEN_INCOME"
vec_base[vec_base=="p3v9_SALARY2"] <- "p3v9_SALARY"
vec_base[vec_base==
           "p17v28_SEX_PART_HAVE_OTHER2"] <- "p17v28_SEX_PART_HAVE_OTHER"
vec_base[vec_base==
           "p17v28_SEX_PART_HAVE_OTHER3"] <- "p17v28_SEX_PART_HAVE_OTHER"

fm1_base <-as.formula(paste("Surv(months,HIV) ~",
                            paste(vec_base, collapse = "+")))
# Model 1 results
cox1_base<- coxph(fm1_base, data =  Base_surv_analysis16.2)

test.ph_base <- cox.zph(cox1_base) # testing for cox ph assumption
test.ph.table_base <- as.data.frame(test.ph_base$table)
test.ph.table.sig_base <- test.ph.table_base[!(test.ph.table_base$p<0.05),]

# Exporting to latex
combine_base <- as.data.frame(cbind(
  summary(cox1_base)$coefficient, summary(cox1_base)$conf.int))
names(combine_base) <- c("coef", "HR", "SE", "Z",
                         "p_value", "SEE","SEEE", "lower", "upper")
combine_base1 <- subset( combine_base, select= c(HR, SE, lower, upper, p_value))
combine_base1 %>% kbl(caption = "Example1", format = "latex", digits = 4)
# Extract AIC value from model 1
extractAIC(cox1_base)
```

Model 2

```
# Calculating mean of the Cytokine profile
Cyt_mean <- Cytokines %>% subset(select = -c(DFSEQ))%>% na.omit() %>%
  group_by(PID) %>%
  summarise_all("mean")

# stepwise Cox regression
Surv_list3<- list(Base_surv_analysis16.1, Cyt_mean)
Base_surv_analysis18<- Surv_list3 %>% reduce(inner_join, by='PID')

Base_surv_analysis18.1 <- subset(Base_surv_analysis18,
                                 select = -c(PID))
Mean_base_Cyt_results <- stepwiseCox(formula=form1,
                                     data = Base_surv_analysis18.1,
                                     selection = "bidirection",
                                     select = "AIC",
                                     sle = 0.15,
                                     sls = 0.15,
                                     method = "efron")
# Using the selected variables from the stepwise regression
vec_mean_base <- as.vector(rownames(Mean_base_Cyt_results$
                                      `Coefficients of the Selected Variables`))
vec_mean_base[vec_mean_base=="Treat2"] <- "Treat"
vec_mean_base[vec_mean_base==
                "p19v14_ABNORMAL_DISCHARGE2"] <- "p19v14_ABNORMAL_DISCHARGE"
vec_mean_base[vec_mean_base=="p3v9_SALARY2"] <- "p3v9_SALARY"
vec_mean_base[vec_mean_base==
                "p18v15_FREQ_CONDOM_USE2"] <- "p18v15_FREQ_CONDOM_USE"
vec_mean_base[vec_mean_base==
                "p17v28_SEX_PART_HAVE_OTHER2"] <- "p17v28_SEX_PART_HAVE_OTHER"
vec_mean_base[vec_mean_base==
                "p17v28_SEX_PART_HAVE_OTHER3"] <- "p17v28_SEX_PART_HAVE_OTHER"
vec_mean_base[vec_mean_base=="p3v8_SELF_GEN_INCOME2"] <- "p3v8_SELF_GEN_INCOME"

fm2 <-as.formula(paste("Surv(months,HIV) ~",
                       paste(vec_mean_base, collapse = "+")))
# Model 2 results
cox2_mean.cyt<- coxph(fm2, data =  Base_surv_analysis18.1)

# testing for Cox PH assumption
test.ph2 <- cox.zph(cox2_mean.cyt)
test.ph.table_2 <- as.data.frame(test.ph2$table)
test.ph.table.sig_2 <- test.ph.table_2[!(test.ph.table_2$p<0.05),]

# Exporting to latex
combine2.1 <- as.data.frame(cbind(
  summary(cox2_mean.cyt)$coefficient, summary(cox2_mean.cyt)$conf.int))
names(combine2.1) <- c("coef", "HR", "SE", "Z",
                       "p_value", "SEE","SEEE", "lower", "upper")
combine2.3 <- subset( combine2.1, select= c(HR, SE, lower, upper, p_value))
combine2.3 %>% kbl(caption = "Example1", format = "latex", digits = 4)
```

```r
#Extracting AIC value for Model 2
extractAIC(cox2_mean.cyt)
```

Model 3

```r
# Calculating the difference for Cytokine Profile
cyt_data <- data.frame(matrix(nrow = 0, ncol = 50))
colnames(cyt_data) <- c(names(Cytokines1))

for (r in unique(Cytokines1$PID)) {
  temp <- Cytokines1 %>% filter(Cytokines1$PID %in% r)
  if(dim(temp)[1] > 1){
  tempnew <- temp[dim(temp)[1],] - temp[1,]
  #print(dim(temp)[1])
  }else{
  tempnew <- temp
  }
  cyt_data[r, ] <- tempnew

}

cyt_data2 <- cyt_data[which(rowMeans(!is.na(cyt_data)) > 0.5), ]
Cytokines_diff<- cyt_data2 %>% subset(select=-c(PID, Follow_up_Month)) %>%
  tibble::rownames_to_column("PID")

Cytokines_diff$PID<-as.numeric(Cytokines_diff$PID)

Surv_list2<-list(HIV1, Cytokines_diff)
Surv_data_complete2<-Surv_list2 %>% reduce(inner_join, by='PID')

# Stepwise Cox regression
Surv_list6<- list(Base_surv_analysis16.1, Cytokines_diff)
Base_surv_analysis21<- Surv_list6 %>% reduce(inner_join, by='PID')
Base_surv_analysis21.1 <- subset(Base_surv_analysis21,
                              select = -c(PID))
Diff_Cyt_base_results <- stepwiseCox(formula=form1,
                             data = Base_surv_analysis21.1,
                             selection = "bidirection",
                             select = "AIC",
                             sle = 0.15,
                             sls = 0.15,
                             method = "efron")
# Using the selected variables from the stepwise regression
vec_diff_base <- as.vector(rownames(Diff_Cyt_base_results$
                                      `Coefficients of the Selected Variables`))
vec_diff_base[vec_diff_base=="Treat2"] <- "Treat"
vec_diff_base[vec_diff_base==
                "p17v28_SEX_PART_HAVE_OTHER2"] <- "p17v28_SEX_PART_HAVE_OTHER"
vec_diff_base[vec_diff_base==
                "p17v28_SEX_PART_HAVE_OTHER3"] <- "p17v28_SEX_PART_HAVE_OTHER"
vec_diff_base[vec_diff_base=="p3v10_HUSBAND2"] <- "p3v10_HUSBAND"
vec_diff_base[vec_diff_base=="p3v9_SALARY2"] <- "p3v9_SALARY"
```

```
vec_diff_base[vec_diff_base=="p3v8_SELF_GEN_INCOME2"] <- "p3v8_SELF_GEN_INCOME"

fm4 <-as.formula(paste("Surv(months,HIV) ~",
                       paste(vec_diff_base, collapse = "+")))
# Model 3 results
cox2_diff.cyt<- coxph(fm4, data =  Base_surv_analysis21.1)

# testing for Cox PH assumptions
test.ph4 <- cox.zph(cox2_diff.cyt) # testing for cox ph assumption
test.ph.table_4 <- as.data.frame(test.ph4$table)
test.ph.table.sig_4 <- test.ph.table_4[!(test.ph.table_4$p<0.05),]

# Exporting to Latex
combine4.1 <- as.data.frame(cbind(
  summary(cox2_diff.cyt)$coefficient, summary(cox2_diff.cyt)$conf.int))
names(combine4.1) <- c("coef", "HR", "SE", "Z",
                       "p_value", "SEE","SEEE", "lower", "upper")
combine4.3 <- subset( combine4.1, select= c(HR, SE, lower, upper, p_value))
combine4.3 %>% kbl(caption = "Example1", format = "latex", digits = 4)

# Extracting AIC value for Model 3
extractAIC(cox2_diff.cyt)
```

Model 4

```
# reformatting data into count process using tmerge function
df_time_ind <-
  tmerge(data1=HIV1,
         data2=HIV1,
         id=PID,
         event=event(months, HIV))
df_final <-
tmerge(data1=df_time_ind,
       data2=Cytokines1,
       id=PID,
       BASIC_FGF=tdc(Follow_up_Month, BASIC_FGF),
       EOTAXIN=tdc(Follow_up_Month, EOTAXIN),
       G_CSF=tdc(Follow_up_Month, G_CSF),
       GM_CSF=tdc(Follow_up_Month, GM_CSF),
       IFN_G=tdc(Follow_up_Month, IFN_G),
       IL_10=tdc(Follow_up_Month, IL_10),
       IL_12P70=tdc(Follow_up_Month, IL_12P70),
       IL_13=tdc(Follow_up_Month, IL_13),
       IL_15=tdc(Follow_up_Month, IL_15),
       IL_17A=tdc(Follow_up_Month, IL_17A),
       IL_1B=tdc(Follow_up_Month, IL_1B),
       IL_1RA=tdc(Follow_up_Month, IL_1RA),
       IL_2=tdc(Follow_up_Month, IL_2),
       IL_4=tdc(Follow_up_Month, IL_4),
       IL_5=tdc(Follow_up_Month, IL_5),
       IL_6=tdc(Follow_up_Month, IL_6),
       IL_7=tdc(Follow_up_Month, IL_7),
```

```
       IL_8=tdc(Follow_up_Month, IL_8),
       IL_9=tdc(Follow_up_Month, IL_9),
       IL_10=tdc(Follow_up_Month, IL_10),
       MCP_1=tdc(Follow_up_Month, MCP_1),
       MIP_1A=tdc(Follow_up_Month, MIP_1A),
       MIP_1B=tdc(Follow_up_Month, MIP_1B),
       PDGF_BB=tdc(Follow_up_Month, PDGF_BB),
       RANTES=tdc(Follow_up_Month, RANTES),
       TNF_A=tdc(Follow_up_Month, TNF_A),
       VEGF=tdc(Follow_up_Month, VEGF),
       CTACK=tdc(Follow_up_Month, CTACK),
       GRO_A=tdc(Follow_up_Month, GRO_A),
       HGF=tdc(Follow_up_Month, HGF),
       IFN_A2=tdc(Follow_up_Month, IFN_A2),
       IL_12P40=tdc(Follow_up_Month, IL_12P40),
       IL_16=tdc(Follow_up_Month, IL_16),
       IL_18=tdc(Follow_up_Month, IL_18),
       IFN_A2=tdc(Follow_up_Month, IFN_A2),
       IL_2RA=tdc(Follow_up_Month, IL_2RA),
       IL_3=tdc(Follow_up_Month, IL_3),
       LIF=tdc(Follow_up_Month, LIF),
       M_CSF=tdc(Follow_up_Month, M_CSF),
       MCP_3=tdc(Follow_up_Month, MCP_3),
       MIF=tdc(Follow_up_Month, MIF),
       MIG=tdc(Follow_up_Month, MIG),
       SCF=tdc(Follow_up_Month, SCF),
       SCGF_B=tdc(Follow_up_Month, SCGF_B),
       SDF_1A=tdc(Follow_up_Month, SDF_1A),
       TNF_B=tdc(Follow_up_Month, TNF_B),
       TRAIL=tdc(Follow_up_Month, TRAIL),
       B_NGF=tdc(Follow_up_Month, B_NGF)
       )
df_final<- df_final %>% na.omit()

# Time dependent Cox formulae
form3 = Surv(tstart, tstop, event) ~ .

# stepwise Cox regression
Base_surv_analysis16.1.1 <-subset(Base_surv_analysis16.1,
                                  select = -c(Treat, Site, months, HIV))
Surv_list9.1<- list(Base_surv_analysis16.1.1, df_final)
Base_surv_analysis23_2<- Surv_list9.1 %>% reduce(inner_join, by='PID') %>%
  na.omit()
Base_surv_analysis23.2 <- subset(Base_surv_analysis23_2,
                             select = -c(PID, months, HIV))

Time_Cyt_base_results.2 <- stepwiseCox(formula=form3,
                          data = Base_surv_analysis23.2,
                          selection = "bidirection",
                          select = "AIC",
                          sle = 0.15,
                          sls = 0.15,
                          method = "efron")
```

```
# Using the selected Variables from the stepwise regression
vec_time_base1 <- as.vector(rownames(
  Time_Cyt_base_results.2$`Coefficients of the Selected Variables`))
vec_time_base1[vec_time_base1==
                 "p3v13_OTHER_INCOME_SOURCE2"] <- "p3v13_OTHER_INCOME_SOURCE"
vec_time_base1[vec_time_base1==
                 "p19v14_ABNORMAL_DISCHARGE2"] <- "p19v14_ABNORMAL_DISCHARGE"
vec_time_base1[vec_time_base1=="p3v9_SALARY2"] <- "p3v9_SALARY"
vec_time_base1[vec_time_base1=="Site2"] <- "Site"

fm6.1 <-as.formula(paste("Surv(tstart,tstop, event) ~",
                         paste(vec_time_base1, collapse = "+")))

# Model 4 results
cox2_time.cyt1<- coxph(fm6.1, data =  Base_surv_analysis23.2)

# Testing for Cox PH assumption
test.ph6.1 <- cox.zph(cox2_time.cyt1) # testing for cox ph assumption
test.ph.table_6.1 <- as.data.frame(test.ph6.1$table)
test.ph.table.sig_6.1 <- test.ph.table_6.1[!(test.ph.table_6.1$p>0.05),]

# Exporting to Latex
combine6.1.1 <- as.data.frame(cbind(
  summary(cox2_time.cyt1)$coefficient, summary(cox2_time.cyt1)$conf.int))
names(combine6.1.1) <- c("coef", "HR", "SE", "Z",
                         "p_value", "SEE","SEEE", "lower", "upper")
combine6.3.1 <- subset( combine6.1.1, select= c(HR, SE, lower, upper, p_value))
combine6.3.1 %>% kbl(caption = "Example1", format = "latex", digits = 4)

# Extract AIC value for Model 4
extractAIC(cox2_time.cyt1)
```

Model graphs

```
## Figure 4
mm1 <- survfit(Surv(months,HIV) ~ 1, data = Base_surv_analysis16.2) %>%
  ggsurv(mm1, surv.col = "black", cens.col = "blue")+
  labs( x="Time (Months)", y="Survival probability", title = "Model 1")+
  theme_bw()

mm2 <- survfit(Surv(months,HIV) ~ 1, data = Base_surv_analysis18.1) %>%
  ggsurv(mm2, surv.col = "black", cens.col = "blue")+
  labs( x="Time (Months)", y="Survival probability", title = "Model 2")+
  theme_bw()

mm3 <- survfit(Surv(months,HIV) ~ 1, data = Base_surv_analysis21.1)%>%
  ggsurv(mm3, surv.col = "black", cens.col = "blue")+
  labs( x="Time (Months)", y="Survival probability", title = "Model 3")+
  theme_bw()
mm4 <- survfit(Surv(tstart, tstop, event) ~ 1, data = Base_surv_analysis23.2)%>%
  ggsurv(mm4, surv.col = "black", cens.col = "blue")+
  labs( x="Time (Months)", y="Survival probability", title = "Model 4")+
```

```r
  theme_bw()
mm_plots <- mm1+mm2+mm3+mm4

ggsave("Figure4.pdf", plot = mm_plots, width = 6, height = 4,
       units = "in", dpi = 600, device = "pdf")
```

```r
## Figure 5
# Model 4 variables trend graph

M4_trend <-aareg(fm6.1, data =  Base_surv_analysis23.2)
M4_trend_plot <- autoplot(M4_trend) + theme_bw()+ theme(legend.position="none")

ggsave("Figure5.pdf", plot = M4_trend_plot, width = 11.5, height = 8.5,
       units = "in", dpi = 600, device = "pdf")
```

```r
## Figure 6
all_model_cyt<- c("SCF", "IL_12P70", "IL_16", "B_NGF", "SCGF_B", "TNF_A", "IL_17A", "CTACK", "IL_10", "[illegible]

fm_m4 <-as.formula(paste("Surv(tstart, tstop, event) ~",
                       paste(all_model_cyt, collapse = "+")))
All_trend_cytokines <- aareg(fm_m4, data =  Base_surv_analysis23.2)
All_trend_cyt_plot <- autoplot(All_trend_cytokines) + theme_bw()+
  theme(legend.position="none")

ggsave("Figure6.pdf", plot = All_trend_cyt_plot, width = 11.5, height = 8.5,
       units = "in", dpi = 600, device = "pdf")
```
